# **Leaf scale quantification of the effect of photosynthetic gas exchange on** Δ_47_**of CO**_**2**_

**DOI:** 10.1038/s41598-021-93092-0

**Published:** 2021-07-07

**Authors:** Getachew Agmuas Adnew, Magdalena E. G. Hofmann, Thijs L. Pons, Gerbrand Koren, Martin Ziegler, Lucas J. Lourens, Thomas Röckmann

**Affiliations:** 1 Institute for Marine and Atmospheric Research Utrecht, Utrecht University, Utrecht, The Netherlands; 2 Institute of Environmental Biology, Utrecht University, Utrecht, The Netherlands; 3 Meteorology and Air Quality Group, Wageningen University, Wageningen, The Netherlands; 4 Department of Earth Sciences, Utrecht University, Utrecht, The Netherlands; 5 Present Address: Picarro B.V., ’s-Hertogenbosch, The Netherlands

**Keywords:** Climate sciences, Environmental sciences

## Abstract

The clumped isotope composition (Δ_47_, the anomaly of the mass 47 isotopologue relative to the abundance expected from a random isotope distribution) of CO_2_ has been suggested as an additional tracer for gross CO_2_ fluxes. However, the effect of photosynthetic gas exchange on Δ_47_ has not been directly determined and two indirect/conceptual studies reported contradicting results. In this study, we quantify the effect of photosynthetic gas exchange on Δ_47_ of CO_2_ using leaf cuvette experiments with one C_4_ and two C_3_ plants. The experimental results are supported by calculations with a leaf cuvette model. Our results demonstrate the important roles of the Δ_47_ value of CO_2_ entering the leaf, kinetic fractionation as CO_2_ diffuses into, and out of the leaf and CO_2_–H_2_O isotope exchange with leaf water. We experimentally confirm the previously suggested dependence of Δ_47_ of CO_2_ in the air surrounding a leaf on the stomatal conductance and back-diffusion flux. Gas exchange can enrich or deplete the Δ_47_ of CO_2_ depending on the Δ_47_ of CO_2_ entering the leaf and the fraction of CO_2_ exchanged with leaf water and diffused back to the atmosphere, but under typical ambient conditions, it will lead to a decrease in Δ_47_.

## Introduction

The carbon dioxide (CO_2_) concentration in the atmosphere is controlled by various large exchange fluxes with the bio-, hydro- and geosphere, and by anthropogenic emissions. Important tools for quantifying the different terms of the budget are measurement of the mole fraction and the isotopic composition of CO_2_^1^. The stable carbon isotope composition allows to distinguish between the CO_2_ uptake by the ocean and by plants^2^, and the stable oxygen isotope composition allows to determine the magnitude of the large gross carbon fluxes between the atmosphere and biosphere^3–6^. Recent advancements in precise measurement techniques of ^17^O-excess^7^ enable the ^17^O-excess, Δ^17^O, of tropospheric CO_2_ to be used as tracer of terrestrial gross primary production^8–13^ or stratospheric influx^14–16^. In addition, the analysis of radiocarbon has been used to quantify the amount of anthropogenic CO_2_ emissions^17,18^. Despite these four independent isotopic tracers, the CO_2_ budget still remains uncertain. It has been suggested that the abundance of the isotopologue ^18^O^13^C^16^O in the atmosphere might be a promising new tracer to complement the existing isotope tracers^19–23^.

The abundance of the double substituted (i.e., contain two rare isotopes) isotopologue ^13^C^18^O^16^O, compared to its abundance at stochastic isotope distribution for a given bulk composition, is also referred to as the clumped isotopic composition of CO_2_ (Δ_47_, see Sect. “Theory” for definition). One of the main reasons for its applicability as a tracer to constrain CO_2_ fluxes is that it is mainly sensitive to the temperature at which the CO_2_ is formed or exchanges isotopes with water^19^. A disadvantage of using Δ_47_ is that signals are very small: For typical ambient surface temperatures of 5 to 30 °C, the thermodynamic equilibrium value of Δ_47_ ranges between 0.90 to 1.04 ‰^19,24,25^, so high precision measurements are required. Thermodynamic equilibrium values of Δ_47_ decrease towards 0 at increasingly higher temperatures, and it is possible to distinguish high temperature (e.g. combustion) from low temperature process (e.g. respiration) using Δ_47_^19,20,22,26^.

It is well established that oxygen isotope exchange between CO_2_ and water in the biosphere and hydrosphere is the dominant process controlling the oxygen isotope composition of atmospheric CO_2_^27,28^. The CO_2_–H_2_O exchange in soils and leaf water is catalyzed by the enzyme carbonic anhydrase (CA)^27,29–31^. In the leaf, CO_2_–H_2_O exchange takes place in the mesophyll, and the mole fraction of the CO_2_ at the CO_2_–H_2_O exchange site is expressed as *c*_m_ (see Sects. “CO_2_ exchange fluxes during photosynthesis” and “Mesophyll conductance”). Laboratory studies have shown that the rate of exchange between CO_2_ and water is the same for δ^18^O and Δ_47_^32,33^. This finding suggests that the equilibration between CO_2_ and leaf water, soil water or open surface water should affect Δ_47_ with similar kinetics as δ^18^O, but in contrast to δ^18^O, Δ_47_ does not depend on the isotopic composition of the different water pools. The rapid isotope exchange of CO_2_ with leaf and surface waters is thought to drive the Δ_47_ of atmospheric CO_2_ towards the thermodynamic equilibrium value^1,19^.

Measurements of Δ_47_ in CO_2_ from air samples show that the clumped isotopic composition in the atmosphere is not in thermodynamic equilibrium at the global mean air temperature. For instance, background CO_2_ from remote air observatories shows Δ_47_ values of 0.92 ‰^21^, which is 0.06 ‰ lower than the expected value of 0.98 ‰ for a global mean air temperature of 15°C^34^. In urban and suburban air, Δ_47_ values are generally even lower and the variability of the reported Δ_47_ values was higher, possibly due to input from anthropogenic CO_2_ formed in high temperature combustion. In Pasadena, Affek and Eiler^20^, Affek, et al.^26^, found Δ_47_ values between 0.73 and 1.01 ‰, and Eiler and Schauble^19^ reported even lower values between 0.62 and 0.93 ‰ from a similar location. Laskar and Liang^22^ reported Δ_47_ values between 0.75 and 0.93 ‰ for urban and suburban air in Taiwan.

Eiler and Schauble^19^ developed a conceptual model to mathematically describe the effect of air-leaf interaction on the Δ_47_ signature of atmospheric CO_2_. The main assumption is that the carbonic anhydrase catalyzed exchange between CO_2_ and H_2_O within the mesophyll will imprint a Δ_47_ value that reflects the effect of leaf temperature on the CO_2_ that diffuses back out of the stomata to the atmosphere. In addition, kinetic fractionation during the diffusion into and out of the leaf through the stomata affects Δ_47_ during photosynthetic gas exchange. Eiler and Schauble^19^ proposed that this kinetic isotope fractionation is significant especially for plant species that show a low carbonic anhydrase activity. In this case, the diffusive component during photosynthesis might lead to a depletion in Δ_47_ of atmospheric CO_2_ of about 0.1 ‰ relative to the thermodynamic equilibrium value of CO_2_–H_2_O exchange alone.

Surprisingly, a recent study, Laskar and Liang^22^ reported Δ_47_ measurements of CO_2_ sampled in a greenhouse that show enrichment by up to 0.08 ‰ in Δ_47_ relative to the thermodynamic equilibrium value. The authors attributed this deviation to kinetic effects associated with the photosynthetic exchange, but as described in Eiler and Schauble^19^, such a kinetic effect should lead to lower, not higher Δ_47_ values relative to the thermodynamic equilibrium. Furthermore Laskar and Liang^22^ concluded that photosynthetic gas exchange would decouple Δ_47_ and δ^18^O in contradiction to the simple CO_2_–H_2_O exchange model of Eiler and Schauble^19^. This discrepancy calls for controlled air-leaf gas exchange experiments to characterize the effect of photosynthesis on Δ_47_ in detail.

Here, we report results from photosynthetic gas exchange experiments under controlled conditions to quantify the effect of gas exchange on the isotopic composition of CO_2_. We investigated the effect on Δ_47_ of the residual CO_2_ (*i*) for different photosynthetic pathways (C_3_ vs. C_4_ plants), (*ii*) for two different values of leaf conductance in C_3_ plants, and (*iii*) for variations in light intensities for one C_3_ plant. This choice of plant species and gas exchange conditions enable us to directly test the proposed hypothesis proposed by Eiler and Schauble^19^ that CO_2_–H_2_O exchange and kinetic fractionation with back-diffusion of CO_2_ to the atmosphere are the main drivers controlling the Δ_47_ fractionation effect of photosynthetic gas exchange.

## Materials and methods

### Theory

#### Background

Δ_47_ describes the deviation of the abundance of ^13^C^18^O^16^O (the dominant isotopologue with nominal mass 47) from the random distribution of all isotopes across all isotopologues in a CO_2_ sample with the same bulk isotopic composition^24^. There are three isotopologues of CO_2_ with nominal mass of 47, ^13^C^18^O^16^O (abundance = 46 × 10^–6^), ^17^O^12^C^18^O (abundance = 1.6 × 10^–6^) and ^17^O^13^C^17^O (abundance = 1.6 × 10^–9^)^19^. The existing isotope ratio mass spectrometer instruments do not have sufficient mass resolving power to separate these isotopologues. Thus, the measured isotope ratio for nominal mass of 47 is a combination of these three isotopologues. Nevertheless, approximately 97% of the CO_2_ with nominal mass 47 is ^13^C^16^O^18^O and the Δ_47_ value is mostly referred to as the value of ^13^C^16^O^18^O isotopologue. Δ_47_ is calculated as^19,35^:

1 $$\Delta _{{47}} = \frac{{^{{47}} R}}{{^{{47}} R^{*} }} - 1$$

where *R* stands for the measured ratio of a rare isotopologue of the indicated mass to the most abundant isotopologue, and R^*^ is the isotopologue abundance ratio assuming that the heavy isotopes are distributed stochastically over all isotopologues^19,24^. In this case, ^47^R^*^ can be calculated from the isotopologues of mass 44 and 47 as ^47^R* = [47]*/[44]* where [44]* = [^12^C][^16^O][^16^O] and 47 = 2[^13^C][^16^O][^18^O] + 2[^12^C][^17^O][^18^O] + [^13^C][^17^O][^17^O]. Note that the factor 2 is a symmetry number. This leads to

2 $${}_{}^{{47}} R^{*} = \frac{{2\left[ {{}_{{\text{}}}^{{13}} {\text{C}}} \right]\left[ {{}_{{\text{}}}^{{16}} {\text{O}}} \right]\left[ {{}_{{\text{}}}^{{18}} {\text{O}}} \right] + \left[ {{}_{{\text{}}}^{{13}} {\text{C}}} \right]\left[ {{}_{{\text{}}}^{{17}} {\text{O}}} \right]\left[ {{}_{{\text{}}}^{{17}} {\text{O}}} \right] + 2\left[ {{}_{{\text{}}}^{{12}} {\text{C}}} \right]\left[ {{}_{{\text{}}}^{{17}} {\text{O}}} \right]\left[ {{}_{{\text{}}}^{{18}} {\text{O}}} \right]}}{{\left[ {{}_{{\text{}}}^{{12}} {\text{C}}} \right]\left[ {{}_{{\text{}}}^{{16}} {\text{O}}} \right]\left[ {{}_{{\text{}}}^{{16}} {\text{O}}} \right]}} = 2{}_{}^{{13}} R{}_{}^{{18}} R + 2{}_{}^{{17}} R{}_{}^{{18}} R + {}_{}^{{13}} R\left( {{}_{}^{{17}} R} \right)^{2}$$

Measurements of both ^13^*R* and ^18^*R* (ratios ^13^C/^12^C and ^18^O/^16^O) require solving Eq. 3.

3 $$- 3K^{2} \left( {{}_{{\text{}}}^{{18}} R} \right)^{{2\lambda }} + 2K{}_{{\text{}}}^{{45}} R\left( {{}_{{\text{}}}^{{18}} R} \right)^{\lambda } + 2{}_{{\text{}}}^{{18}} R - {}_{{\text{}}}^{{46}} R =0$$

where *K* is ^17^R_std_/(^18^R_std_) ^*λ*^^36^ and *λ* is the three isotope exponent.

^13^*R* and ^18^*R* can be calculated from the corresponding δ values as ($^{{13}} R = \left( {\delta ^{{13}} {\text{C}}_{{{\text{VPDB}}}} + 1} \right) \times 0.011180$, and $^{{18}} R = \left( {\delta ^{{18}} {\text{O}}_{{{\text{VSMOW}}}} + 1} \right) \times 0.0020052$). It is impossible to measure ^17^*R* of CO_2_ directly with traditional gas source isotope ratio mass spectrometry due to the isobaric interference of ^13^C^16^O^16^O on ^12^C^17^O^16^O. Variations in the isotope ratios ^18^*R* and ^17^*R* in a sample are closely linked in most common processes via the mass dependent fractionation equation [^18^*R*/^18^*R*_std_]^*λ*^ = ^17^*R*/^17^*R*_std_ where *std* stands for standard^36^. In this study, a value of 0.528 is used as recommended by^37,38^. ^17^*R* was calculated as (${}_{{}}^{{17}} R = \left( {{}_{{\text{~}}}^{{18}} {\text{R}}/0.0020052} \right)^{{0.528}} \times 0.0003799$)^35,37,38^. Recently, we have shown that ^17^*R* can be measured independent of ^13^C interference on O fragment ions^7^.

δ^13^C and δ^18^O of the sample are calculated from δ^45^ and δ^46^ (i.e. δ^13^C_VPDB_ ≅ δ^45^_sample_ + 2 × (^17^R/^13^R)_VPDB-CO2_ × ( δ^45^_sample_ − λ × δ^45^_sample_) and δ^18^O_VPDB-CO2_ ≅ [ δ^46^_sample_ − 0.0021 × δ^13^C_VPDB_]/0.99904), where ^17^*R*/^13^*R* is 0.03516^37^. The δ value is calculated as follow:

4 $$\delta ^{{\text{x}}} = \left( {\frac{{^{{\text{x}}} {\text{R}}_{{{\text{sample}}}} }}{{^{{\text{x}}} {\text{R}}_{{{\text{standard}}}} }} - 1} \right)$$

where *x* can be 13, 18, 45, 46 and 47 (for ^13^C, ^18^O, ^13^C^16^O^16^O, ^12^C^16^O^18^O and ^13^C^16^O^18^O, respectively).

#### CO_2_ exchange fluxes during photosynthesis

**Figure 1:**
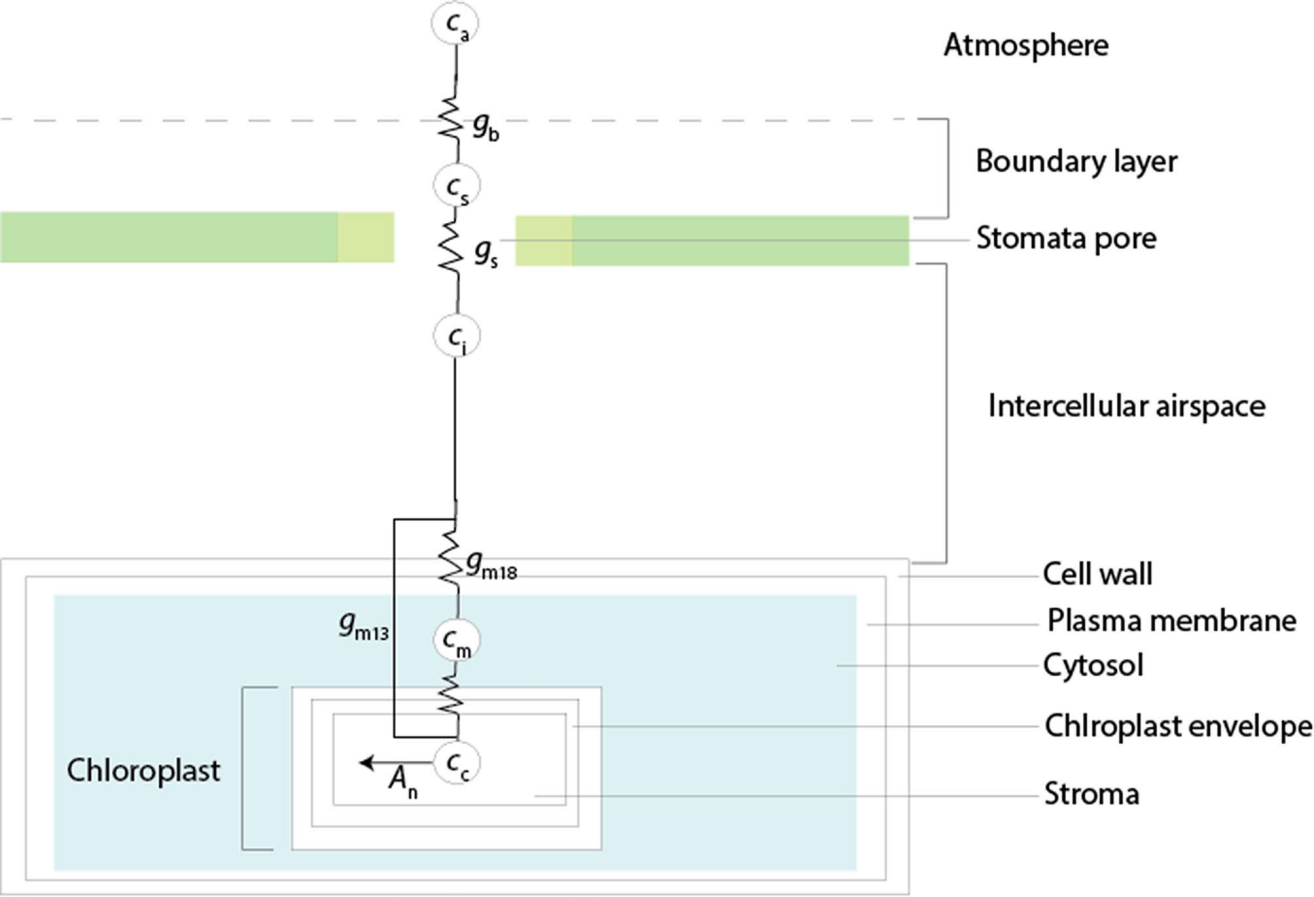
Schematic illustration of CO_2_ exchange fluxes, concentrations and conductivities during photosynthetic gas exchange. Net photosynthetic CO_2_ uptake in a leaf generates a concentration gradient over the leaf where *c*_a_ is the CO_2_ concentration of the air, *c*_s_ the CO_2_ concentration at the leaf surface, *c*_i_ is the CO_2_ concentration in the intercellular air space, *c*_m_ is the CO_2_ concentration in the mesophyll, i.e., the site of CO_2_-H_2_O exchange and *c*_c_ the CO_2_ concentration in the chloroplasts, the site of CO_2_ uptake. The *g*_b_ is the boundary layer conductance of CO_2_ from the atmosphere to the leaf surface, the stomatal conductance *g*_s_ quantifies the gas exchange through the stomatal opening, *g*_m13_ is the mesophyll conductance to the site of carbon uptake and *g*_m18_ is the mesophyll conductance to the site of CO_2_-H_2_O exchange.

The CO_2_ uptake by C_3_ plants is schematically illustrated in Fig. 1. Net photosynthetic CO_2_ uptake in a leaf generates a concentration gradient from the atmosphere (*c*_a_) to the boundary layer (*c*_s_), intercellular airspace (*c*_i_), the mesophyll cell (*c*_m_) and the chloroplast (*c*_c_), where CO_2_ is fixed (in C_3_ plants) (see Fig. 1)^39–42^. In the chloroplast, the enzyme RubisCO catalyzes the conversion of carbon dioxide to the three-carbon acid 3-phosphoglyceric acid (3PGA). *c*_c_ determines the availability of CO_2_ for carboxylation, which is the rate-limiting substrate. The concentration gradient between $c_{{\text{a}}}$ and $c_{\text{c}}$ drives the diffusion of CO_2_ into the leaf. The diffusion process can be described mathematically following Fick’s law of diffusion as:

5 $$A_{{\text{n}}} = g_{{\text{L}}} \left( {c_{{\text{a}}} - c_{{\text{c}}} } \right)$$

where *g*_L_ is the total conductance (inverse of resistance, 1/*r*_L_) of the leaf for CO_2_ diffusion. Total *g*_L_ is conveniently subdivided into three parts that act in series. The boundary layer conductance (*g*_b_) represents the conductance through a thin layer of near-stagnant air surrounding the leaf; it is a function of air turbulence and leaf area. The stomatal conductance (*g*_s_) varies with the opening and frequency of the stomata. The mesophyll conductance (*g*_m_) quantifies the conductance for transport of CO_2_ from the intercellular air space in the leaf to the site of CO_2_–H_2_O exchange in the mesophyll or the carboxylation in the chloroplast. The latter is expressed as *g*_m13_ and the former is denoted as *g*_m18_, which refers to estimation using δ^13^C and δ^18^O respectively (see Sect. “Mesophyll conductance"). A small part of this transport occurs in the gas phase through a residual part of the intercellular air space. The major transport pathway is in the liquid phase, through the wall of the mesophyll cell, the plasmalemma and further into the cell to the site of oxygen exchange between CO_2_ and H_2_O that is catalyzed by carbonic anhydrase (CA). CO_2_ diffuses further through the chloroplast envelope and into the chloroplast where carboxylation occurs^39,40,43^.

#### Mesophyll conductance

For C_3_ plants, we used the discrimination against ^13^C (Δ_A_^13^C) (Eq. 6) to estimate the mesophyll conductance ($g_{{m13}}$) from the intercellular airspace to the carboxylation site (Eq. 7) as described in^44^. The overall isotope fractionation associated with photosynthetic gas exchange is referred to as discrimination, which quantifies the enrichment or depletion of the isotope composition of CO_2_ in the surrounding atmosphere relative to the CO_2_ assimilated, see Eq. (6)^42,45,46^. Experimentally, the discrimination (Δ_A_) is calculated from the isotopic composition and mole fraction of CO_2_ entering and leaving the cuvette^47–49^, as shown in Eq. (6).

6 $$\Delta _{{\text{Aobs}}}^{{{\text{x}} }} = \frac{{\zeta ~\left( {\delta _{{\text{a}}}^{{\text{x}}} - \delta _{{\text{e}}}^{{\text{x}}} } \right)}}{{1 + \delta _{{\text{a}}}^{{\text{x}}} - \zeta \left( {\delta _{{\text{a}}}^{{\text{x}}} - \delta _{{\text{e}}}^{{\text{x}}} } \right)}}$$

where *x* is either 13, 18 or 47 (for ^13^C, ^18^O and ^13^C^18^O^16^O isotope composition, respectively), $\zeta = c_{e} /\left( {c_{e} - c_{a} } \right)$ and* c* is the mole fraction of the CO_2_ entering (index *e*) and leaving (index a) the cuvette.

$g_{{m13}}$ is calculated from the difference between the observed ^13^C discrimination (Δ_A_^13^C_obs_) and the discrimination at infinite *g*_m_ (*c*_i_ = c_c_) as:

7 $${\text{g}}_{{{\text{m}}13}} = \frac{{{\text{A}}_{{\text{n}}} /{\text{P}}}}{{{\text{c}}_{{\text{i}}} - {\text{c}}_{{\text{c}}} }} = \left( {\frac{{1 + {\text{t}}^{{13}} }}{{1 - {\text{t}}^{{13}} }}} \right)\left( {\frac{{{\text{A}}_{{\text{n}}} \left( {{\text{b}} - {\text{a}}_{{\text{m}}} - \frac{{\alpha _{{\text{b}}} }}{{\alpha _{{\text{e}}} \alpha _{{\text{R}}} }}e^{\prime } \frac{{{\text{R}}_{{\text{D}}} }}{{{\text{A}}_{{\text{n}}} }}} \right)}}{{\left( {\Delta _{{\text{A}}}^{{13}} {\text{C}}_{{\text{i}}} - \Delta _{{\text{A}}}^{{13}} {\text{C}}_{{{\text{obs}}}} } \right){\text{Pc}}_{{\text{a}}} }}} \right)$$

where *P* is the ambient pressure, *t*^*13*^ is a ternary correction factor for ^13^CO_2_, *b* the fractionation due to uptake by Rubisco, and *a*_m_ the combination of the fractionations associated with ^13^CO_2_ dissolution and diffusion through water, respectively. *e’*, *R*_D_, $\alpha _{e}$, and $\alpha _{b}$ are the fractionation factor for mitochondrial respiration including the apparent fractionation, the day respiration rate (mitochondrial respiration in the light) the fractionation factor for day respiration with respect to net assimilation and the fractionation factor for C_3_ carboxylation, respectively. $\alpha _{R}$ = 1 + (R_D_/A_n_) × (*e’*/$\alpha _{e}$). A detailed description of the equations, used parameters and definitions of fractionation factors is provided in Table S1 of the supplementary material.

In C_4_ plants, the CO_2_ is converted to bicarbonate and fixed to a four-carbon acid catalyzed by phosphoenol pyruvate carboxylase (PEPC) in the mesophyll. Unlike for C_3_ plants, it is impossible to estimate mesophyll conductance from Δ_A_^13^C for C_4_ plants due to the low photosynthetic fractionation^50,51^. However, the apparent discrimination against ^18^O (Δ_A_^18^O) can be used to estimate the mesophyll conductance from the intercellular air space to the site of CO_2_–H_2_O isotope equilibration, $g_{{m18}}$, as described in^41,47,52–54^, for both C_3_ and C_4_ plants. The analytical expression for estimating $g_{{m18}}$ (Eq. 8) assumes that the degree of equilibration between CO_2_ and H_2_O is 100%. Some studies have reported that the degree of equilibration can be lower than 100%, especially for C_4_ plants, which have a lower CA activity^29,31,47,54^. Detailed information is provided in Table S1 of the supplementary material.

8 $${\text{g}}_{{{\text{m}}18}} = \frac{{{\text{A}}_{{\text{n}}} /{\text{P}}}}{{{\text{c}}_{{\text{i}}} - {\text{c}}_{{\text{m}}} }} = \left( {\frac{{{\text{A}}_{{\text{n}}} /{\text{P}}}}{{{\text{c}}_{{\text{i}}} }}} \right)\frac{{\delta ^{{18}} {\text{O}}_{{\text{A}}} \alpha _{{18{\text{w}}}} + {\text{a}}_{{18{\text{w}}}} - \delta ^{{18}} {\text{O}}_{{\text{m}}} }}{{\delta ^{{18}} {\text{O}}_{\text{i}} - \delta ^{{18}} {\text{O}}_{{\text{m}}} }}$$

δ^18^O_i_ is δ^18^O of CO_2_ in the intercellular airspace, $\alpha _{{18w}}$ is the fractionation factor for ^12^C^18^O^16^O during diffusion and dissolution in water, $a_{{18w}}$ is the discrimination against ^12^C^18^O^16^O during diffusion and dissolution in water, $a_{{18w}} = \alpha _{{18w}} - 1$, δ^18^O_A_ is δ^18^O of the assimilated CO_2_ and δ^18^O_m_ is the δ^18^O of CO_2_ in equilibrium with leaf water at the CO_2_–H_2_O exchange site. In previous studies, the difference between δ^18^O of bulk leaf water and the water at the evaporation site was about 1 to 2 ‰ higher^13,54^, but larger differences have also been reported^55^. In this study, we assumed that the δ^18^O of leaf water at the CO_2_–H_2_O exchange site is enriched by 2 ‰ compared to bulk leaf water and the degree of equilibration between CO_2_ and leaf water is 100%.

#### Photosynthetic Δ_47_ discrimination

**Figure 2:**
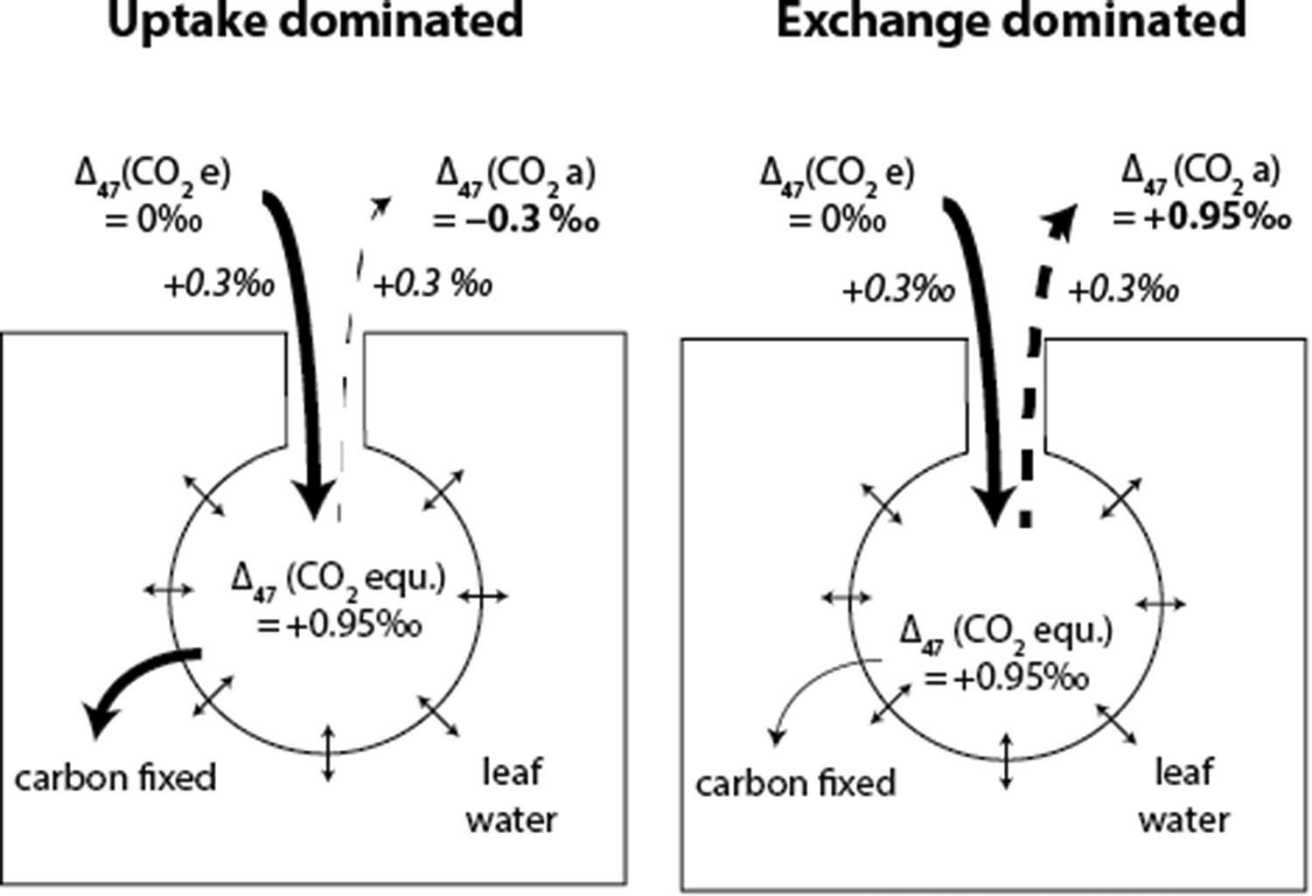
Schematic illustration of the clumped isotope fractionation during air-leaf gas exchange. Δ_47_ of the CO_2_ in the air surrounding the leaf is mainly controlled by CO_2_-H_2_O exchange and kinetic fractionation during diffusion into and out of the leaf stomata. In the uptake dominated case (*c*_m_/*c*_a_ ~ 0), − 0.3 ‰ comes from the fact that diffusion mediated leaf air interaction will reduce the Δ_47_ value by 0.3 ‰, neglecting other effects. Diffusion only will reduce the Δ_47_ value by 0.3 ‰ multiplied by the fraction of CO_2_ entering the leaf that is assimilated ^19^. The boundary layer conductance is large and we have omitted it here.

The effect of photosynthetic gas exchange on Δ_47_ depends on the assimilation rate, the various conductances mentioned before and the fraction of CO_2_ that diffuses back to the atmosphere after isotope exchange with leaf water. For a laminar boundary layer, diffusion through the boundary layer decreases the Δ_47_ value of residual CO_2_ by an amount equal to the boundary layer diffusive fractionation (+ 0.2 ‰) multiplied by the fraction of CO_2_ enters leaves and is fixed (~ 1/3), i.e., 0.07 ‰. In this study, we used a fan (see Sect. “Leaf cuvette set-up”), which creates a turbulent boundary layer with an estimated conductance of 5 mol m^−2^ s^−1^. The boundary layer conductance for the leaf cuvette was determined using wet filter paper from measurements of relative humidity and temperature of air in the cuvette as described in Parkinson ^56^. As a result, the fractionation in Δ_47_ due to diffusion through the boundary layer is negligible compared to the precision of the measurement (ca. ~ 0.002 ‰). Figure 2 illustrates the effect of photosynthetic gas exchange on Δ_47_ for two extreme scenarios. The first “uptake dominated” scenario assumes that all the CO_2_ entering stomata gets assimilated, leading to *c*_m_/*c*_a_ ~ 0 whereas in the second “exchange dominated” scenario all CO_2_ diffuses back to the atmosphere after exchange with the leaf water leading to *c*_m_/*c*_a_ ~ 1. In the uptake dominated scenario, the resulting Δ_47_ signal is mainly affected by diffusion, in the exchange dominated scenario the Δ_47_ signal is dominated by the CO_2_–H_2_O exchange. A similar scheme for δ^18^O is shown in Figure S2 of the supplementary material.

The fractionation of Δ_47_ associated with photosynthetic gas exchange (Δ_A_Δ_47obs_) was calculated in a similar way as $\Delta _{{{\text{Aobs}}}}^{{x}}$ in Eq. (6) from the difference in CO_2_ concentration and isotopic composition between the air entering and leaving the leaf cuvette (Eq. 9). For mixing of two different populations of CO_2_, Δ_47_ is not a conserved quantity and the error introduced by adding or subtracting Δ_47_ values linearly depends on the relative difference in the δ^18^O and δ^13^C of the two gases^19,20,57,58^. In our study, the maximum difference between the δ^18^O and δ^13^C value of the CO_2_ entering and leaving the cuvette is 14 ‰ and 4 ‰, respectively. For this rather small range, the error introduced due to linear addition and subtraction of Δ_47_ values of the CO_2_ entering and leaving the cuvette is not significant (< 0.01 ‰).

9 $$\Delta _{{\text{A}}} \Delta _{{4{\text{7obs}}}} = \frac{{\zeta ~\left( {\Delta _{{47{\text{a}}}} - \Delta _{{47{\text{e}}}} } \right)}}{{1 + \Delta _{{47{\text{a}}}} - \zeta \left( {\Delta _{{47{\text{a}}}} - \Delta _{{47{\text{e}}}} } \right)}}$$

### Leaf cuvette model

To explore the effects of conductance, assimilation rate and back-diffusion of CO_2_ to the atmosphere on the Δ_47_ of ambient CO_2_ in detail we used a leaf cuvette model [https://git.wur.nl/leaf-model]^59^ that has been used for the interpretation of Δ^17^O measurements in leaf exchange experiments recently^13^. In the model we assumed that CO_2_–H_2_O exchange in the mesophyll is rapid enough to constantly set Δ_47_ to thermodynamic equilibrium with the water, i.e., Δ_47_ = 0.95 ‰ at 20 °C^25^. Thus, any change in bulk isotopic composition of CO_2_ due to assimilation does not affect Δ_47_^19^. Furthermore, CO_2_ diffusion into and out of the intercellular air space through the stomata is associated with a fractionation constant of + 0.3 ‰ for Δ_47_^19^ whereas CO_2_ diffusion through the boundary layer has a fractionation constant of + 0.2 ‰ for Δ_47_.

The steady state model considers five compartments: (i) atmosphere (air in the leaf surrounding), (ii) the leaf surface, (iii) the intercellular airspace of the leaf, (iv) the mesophyll cell of the leaf, and (v) the chloroplast (see Fig. 1). The air enters the leaf cuvette at a flow rate *F*_e_ and a CO_2_ concentration *c*_e_ with a well-defined isotopic composition δ_e_ (where δ can stand for δ^13^C, δ^18^O, δ^47^ and Δ_47_, see Sect. “Background” for definition). The leaf inside the cuvette takes up a portion of the CO_2_ and this uptake is associated with an isotope fractionation. The air flowing out of the cuvette has flow rate *F*_a_, CO_2_ concentration *c*_a_ and isotopic composition δ_a_. The photosynthetic uptake in the chloroplasts leads to a concentration gradient between the air surrounding the leaf and the chloroplasts so that there is a net flow of CO_2_ from the cuvette into the intercellular airspace, to the mesophyll cell, and finally to the chloroplasts. The corresponding CO_2_ concentrations decrease accordingly in the order *c*_a_, *c*_s_, *c*_i_, *c*_m_ and *c*_c_. Diffusion, isotopic equilibration with H_2_O, CO_2_ uptake and mixing between the model reservoirs lead to a change in isotopic composition (δ_a_, δ_s_, δ_i_, δ_m_ and δ_c_). The magnitude of the exchange fluxes between the compartments is defined by the boundary layer conductance *g*_b_, stomatal conductance *g*_s_ and the mesophyll conductances *g*_m18_ and *g*_m13_. In the leaf cuvette model, the boundary layer conductance *g*_b_ is assumed 5 mol m^−2^ s^−1^, similar to the value determined in the experiment.

**Figure 3:**
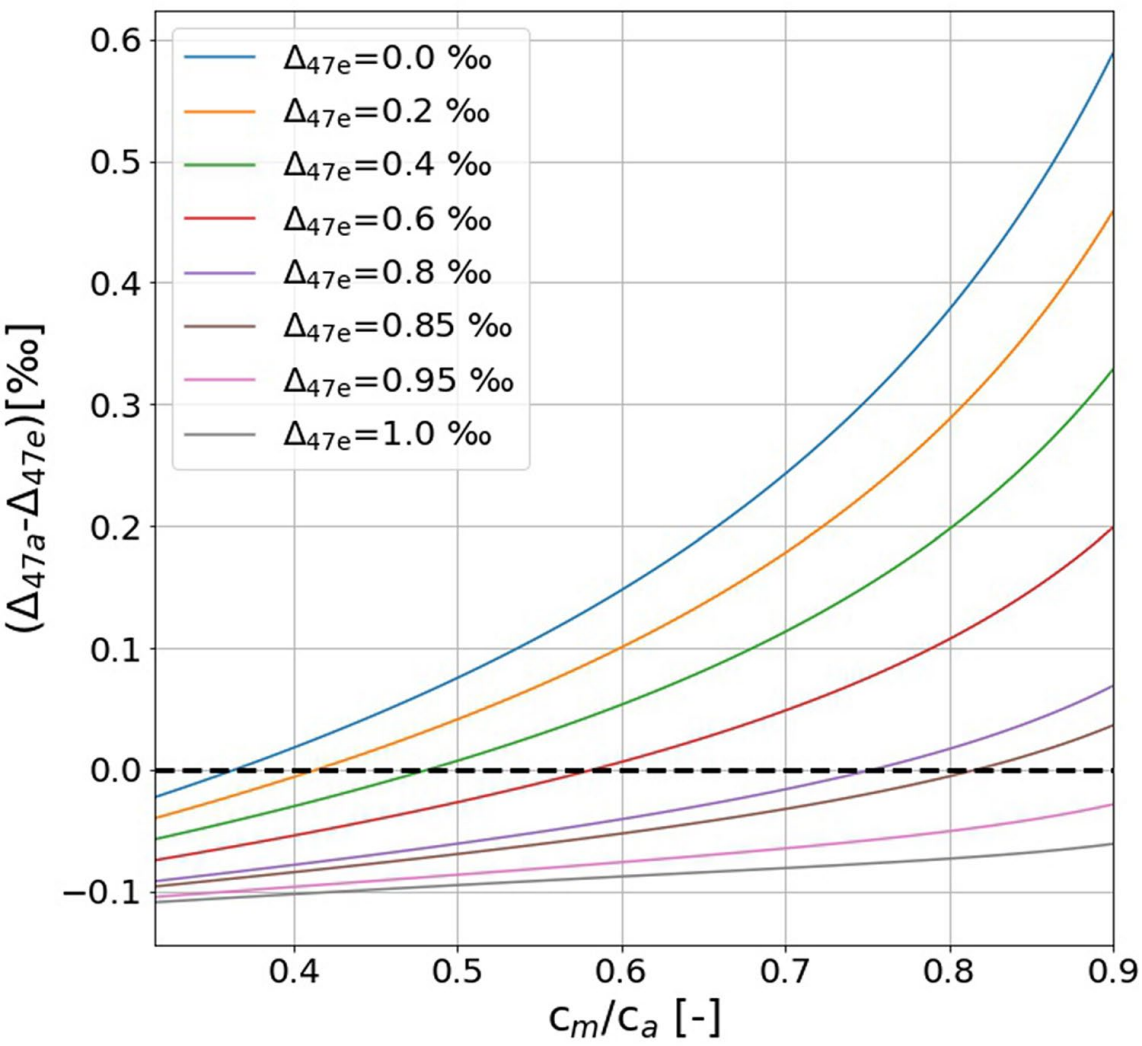
Difference between Δ_47_ of out- and inflowing CO_2_ as function of the fraction of CO_2_ diffusing back to the atmosphere (*c*_m_/*c*_a_) calculated with the leaf cuvette model. The black dashed line shows the *c*_m_/*c*_a_ ratio where the Δ_47_ relative difference between the ingoing and outgoing CO_2_ becomes zero for the corresponding Δ_47_ of CO_2_ entering the cuvette.

Figure 3 shows how the Δ_47_ value changes between incoming and outgoing CO_2_ in the leaf cuvette model for *c*_m_/*c*_a_ ratios ranging from 0.3 to 0.9 and Δ_47_ values of the entering CO_2_ between 0.0 ‰ and 1.0 ‰. It is evident that the relative changes are small when Δ_47_ of the incoming CO_2_ is close to the equilibrium value (0.9—1.0 ‰) at ambient temperatures. The changes are much larger if the Δ_47_ of the incoming CO_2_ is close to a random distribution (Δ_47_ = 0.0 ‰). This motivated us to carry out the gas exchange experiments with isotopically ‘scrambled' (i.e., Δ_47_ close to zero) CO_2_ (see below).

### Plant material and growth conditions

Three plant species were used for the experiments, belonging to different functional groups: Sunflower (*Helianthus annuus* L. cv, Giganteus), Atlantic ivy [*Hedera hybernica* (Kirchner) Bean (syn. *Hedera helix* var. hibernica)], and maize (*Zea mays* L. cv, Torres). The fast-growing annual C_3_ species *Helianthus* is characterized by short-lived leaves with high photosynthetic capacity and conductance for CO_2_ diffusion. The other C_3_ species, the evergreen *Hedera*, has long-lived leaves with a lower photosynthetic capacity and conductance. The third species, *Zea*, has C_4_ metabolism, a high photosynthetic capacity and low conductance.

*Helianthus* and *Zea* were grown from seed in a growth room at 20 °C, a relative humidity of 70% and a photosynthetic photon flux density (PPFD) of 250 µmol m^−2^ s^−1^ for a day length of 16 h. The first pair of two leaves of *Helianthus* was used for the experiments when fully grown at three to four weeks after planting. Younger leaves that shaded them were removed. For *Zea* *mays*, a section of the fifth or sixth leaf at about two thirds of total leaf length was used at a plant age of around 7 weeks. These leaves were about 5 cm wide, giving a sufficiently large leaf area in the cuvette. *Hedera* plants were obtained from a grower, pruned to reduce self-shading and further grown in the experimental garden in full daylight. They were used for the experiments in early November 2015 when outside average maximum day temperature during the preceding month was 14 °C. Mature leaves that could be accommodated intact in the cuvette were used for the experiments. For all the experiments, leaves remained attached to the plants during the experiments. Both *Helianthus* and *Zea* are watered from tap water whereas *Hedera* received largely rain water.

### Leaf cuvette set-up

**Figure 4:**
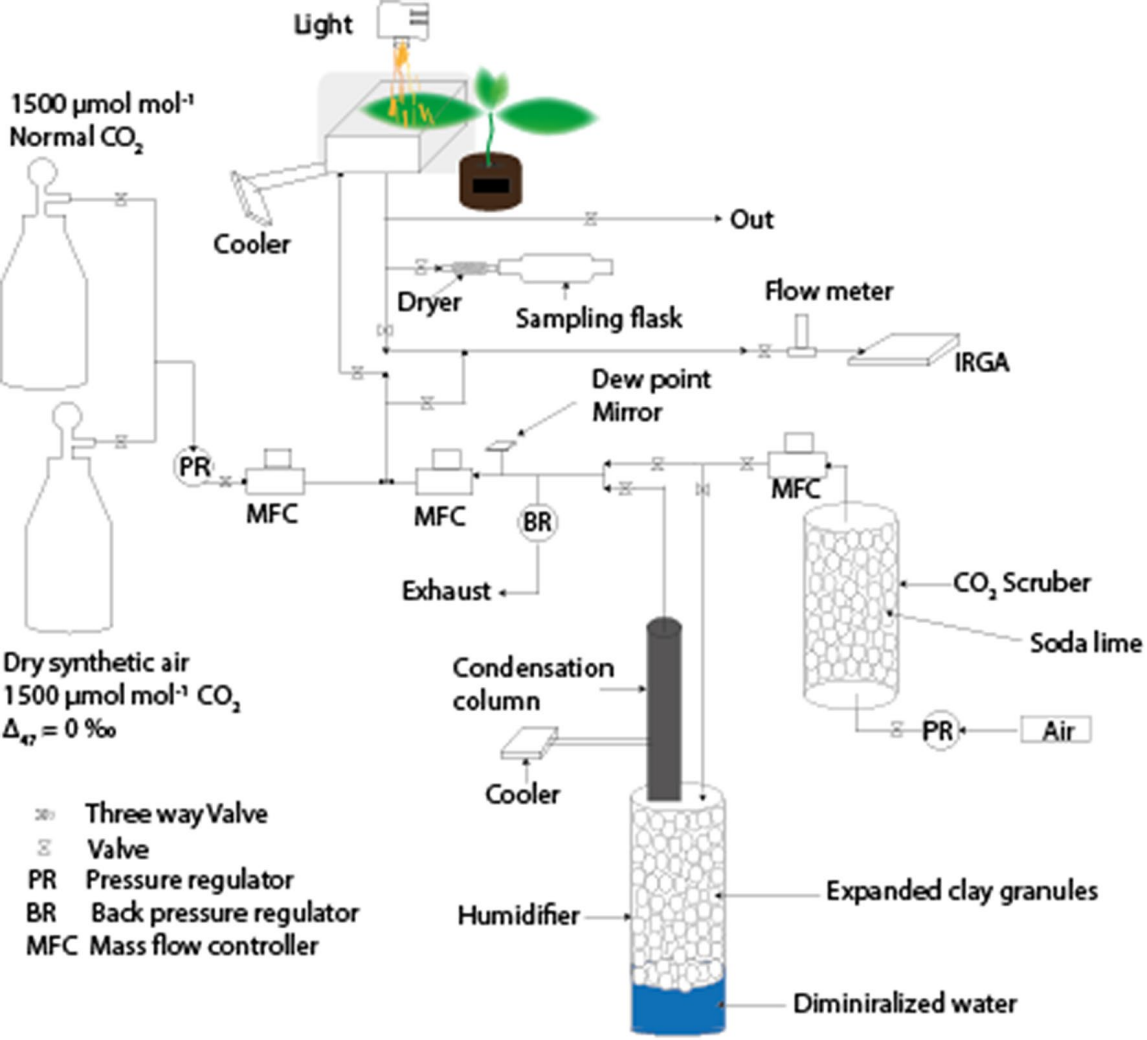
Leaf chamber set-up, modified from Adnew et al. (2020)^13^. A single leaf was placed in the leaf cuvette and the light intensity could be regulated to manipulate the assimilation rate. A gas-mixing unit was used to mix dry synthetic air with scrambled CO_2_ with humidified CO_2_-free air to obtain an overall CO_2_ concentration of the ingoing air of 500 µmol mol^−1^. The flow rate was adjusted to the photosynthetic activity of the leaf to obtain a CO_2_ concentration of about 400 µmol mol^−1^ at the outlet (0.6 to 1.5 L min^−1^). CO_2_ and H_2_O concentrations were monitored with an infrared gas analyzer (IRGA). Once steady state was reached, the outgoing air was sampled in one 2 L and one 1 L glass flasks, in series. The bulk isotope composition (δ^13^C and δ^18^O) of the normal CO_2_ and scrambled CO_2_ are identical.

The isotopic effect of CO_2_ exchange during photosynthesis was investigated with an open gas exchange measurement system similar to the one described by^13,60^ (Fig. 4). A controlled flow of air entered and left the leaf cuvette, which had a 7 × 7 cm transparent window on top that limited the maximum width of the leaves that could be accommodated. A fan inside the leaf cuvette increased boundary layer conductance to around 5 mol m^−2^ s^−1^ and mixed the air thoroughly so that the air leaving the cuvette was a representative sample of the air inside. The chamber was illuminated from above by a halogen lamp that allowed control of the PPFD incident on the leaf. Leaf temperature was measured with thermocouples appressed to the abaxial side of the leaf. Water at 20 °C was circulated through the double wall of the cuvette, which stabilized leaf temperature up to 3 °C higher depending on PPFD and transpiration rate (Table 1).

**Table 1:** Gas exchange variables and isotopic composition of the bulk leaf water. *T*_leaf_: leaf temperature; *c*_e_: CO_2_ concentration of the air entering the leaf cuvette; *c*_a_: CO_2_ concentration of the air inside the cuvette and leaving the cuvette; *A*_n_: net assimilation rate; *E*: transpiration rate; *g*_s_: stomatal conductance for CO_2_; *c*_i_: CO_2_ concentration in the intercellular airspace; *c*_c_: CO_2_ concentration in the chloroplasts (site of CO_2_ uptake); *c*_m_: CO_2_ concentration in the mesophyll cell; *g*_m13_: mesophyll conductance from the intercellular airspace to the chloroplasts; *g*_m18_ : mesophyll conductance from the intercellular airspace to the CO_2_-H_2_O exchange site; δ^18^O_LW_: δ^18^O value of the bulk leaf water vs VSMOW (‰). The values in bold are mean and standard deviation for the replicates at different light conditions.

Species	Leaf type	PPFD $\left[ {\frac{{\mu mol}}{{m^{2} ~s}}} \right]$	No. exp	*T*_leaf_ [°C]	*c*_e_ $\left[ {\frac{{\mu mol}}{{mol}}} \right]$	*c*_a_ $\left[ {\frac{{\mu mol}}{{mol}}} \right]$	*A*_n_ $\left[ {\frac{{\mu mol}}{{m^{2} ~s}}} \right]$	*E* $\left[ {\frac{{mmol}}{{m^{2} ~s}}} \right]$	*g*_s_ $\left[ {\frac{{mol}}{{m^{2} ~s}}} \right]$	*g*_m13_ $\left[ {\frac{{mol}}{{m^{2} ~s~bar}}} \right]$	*g*_m18_ $\left[ {\frac{{mol}}{{m^{2} ~s~bar}}} \right]$	*c*_i_/*c*_a_	*c*_c_/*c*_a_	*c*_*m*_*/c*_*a*_	δ^18^O_LW_
***H.annuus***	**C**_3_	200	(1)	21.4	499	420	12.0	2.3	0.27	0.33 ± 0.05	0.59 ± 0.05	0.80	0.71 ± 0.02	0.75 ± 0.01	4.3 ± 0.1
			(2)	21.2	499	406	12.2	2.6	0.32	0.17 ± 0.05	0.82 ± 0.05	0.81	0.64 ± 0.02	0.78 ± 0.03	6.0 ± 0.1
			(3)	20.6	501	409	11.3	2.1	0.35	0.36 ± 0.05	0.64 ± 0.05	0.85	0.78 ± 0.02	0.81 ± 0.01	6.5 ± 0.1
		**Mean** ± **SD**		**21.1 ± 0.4**	**500 ± 1**	**412 ± 7**	**11.8 ± 0.5**	**2.3 ± 0.3**	**0.31 ± 0.04**	**0.27 ± 0.1**	**0.68 ± 0.12**	**0.82 ± 0.03**	**0.71 ± 0.07**	**0.78 ± 0.03**	**5.6 ± 1.2**
		700	(4)	21.8	500	399	24.4	4.1	0.51	0.61 ± 0.05	0.93 ± 0.05	0.78	0.68 ± 0.02	0.71 ± 0.01	4.7 ± 0.1
			(5)	22.2	499	406	20.9	3.0	0.33	0.46 ± 0.05	0.67 ± 0.05	0.74	0.63 ± 0.02	0.66 ± 0.01	6.1 ± 0.1
			(6)	21.3	500	404	20.8	3.5	0.45	0.43 ± 0.05	0.52 ± 0.05	0.79	0.67 ± 0.02	0.69 ± 0.01	6.6 ± 0.1
			(7)	21.4	499	405	20.7	3.7	0.45	0.65 ± 0.05	0.72 ± 0.05	0.79	0.71 ± 0.02	0.72 ± 0.01	6.5 ± 0.1
		**Mean** ± **SD**		**21.7 ± 0.4**	**500 ± 1**	**404 ± 3**	**21.7 ± 1.8**	**3.6 ± 0.5**	**0.44 ± 0.08**	**0.54 ± 0.1**	**0.71 ± 0.17**	**0.78 ± 0.02**	**0.67 ± 0.03**	**0.70 ± 0.03**	**6.0 ± 0.9**
		1800	(8)	23.7	500	401	27.8	5.4	0.53	0.46 ± 0.05	0.81 ± 0.05	0.74	0.59 ± 0.02	0.66 ± 0.01	6.7 ± 0.1
			(9)	23.8	502	411	22.2	4.5	0.39	0.45 ± 0.05	0.55 ± 0.05	0.73	0.62 ± 0.02	0.64 ± 0.01	6.9 ± 0.1
			(10)	22.9	501	405	23.5	4.5	0.55	0.37 ± 0.05	0.59 ± 0.05	0.79	0.63 ± 0.02	0.69 ± 0.01	5.9 ± 0.1
		**Mean** ± **SD**		**23.5 ± 0.5**	**501 ± 1**	**406 ± 5**	**24.5 ± 2.9**	**4.8 ± 0.5**	**0.49 ± 0.09**	**0.43 ± 0.05**	**0.65 ± 0.14**	**0.75 ± 0.03**	**0.61 ± 0.02**	**0.66 ± 0.03**	**6.5 ± 0.5**
***H.Hibernica***	**C**_3_	700	(1)	22.6	499	404	15.2	1.2	0.08	0.21 ± 0.05	0.36 ± 0.05	0.41	0.23 ± 0.02	0.30	2.4 ± 0.1
			(2)	22.8	500	395	14.8	1.0	0.08	0.17 ± 0.05	0.24 ± 0.05	0.41	0.20 ± 0.02	0.26	1.9 ± 0.1
			(3)	22.7	498	395	15.8	1.3	0.09	0.21 ± 0.05	0.31 ± 0.05	0.42	0.23 ± 0.02	0.29	4.9 ± 0.1
		**Mean** ± **SD**		**22.7 ± 0.1**	**499 ± 1**	**398 ± 5**	**15.3 ± 0.5**	**1.2 ± 0.2**	**0.08 ± 0.01**	**0.20 ± 0.02**	**0.30 ± 0.06**	**0.41 ± 0.01**	**0.22 ± 0.02**	**0.28 ± 0.02**	**3.1 ± 1.6**
***Z. mays***	**C**_4_	700	(1)	22.0	501	404	28.2	1.8	0.11	–	0.65 ± 0.05	0.23	–	0.12	19.3 ± 0.1
			(2)	21.8	499	411	29.2	1.9	0.12	–	0.58 ± 0.05	0.26	–	0.14	20.3 ± 0.1
			(3)	22.0	501	401	26.7	1.7	0.16	–	0.49 ± 0.05	0.48	–	0.35	21.9 ± 0.1
		**Mean** ± **SD**		**21.9 ± 0.1**	**500 ± 1**	**405 ± 5**	**28 ± 1.3**	**1.8 ± 0.1**	**0.13 ± 0.03**		**0.57 ± 0.08**	**0.32 ± 0.14**		**0.20 ± 0.13**	**20.5 ± 1.3**

A gas-mixing unit made from ¼ inch stainless-steel tubing was attached to the inlet port of the cuvette. Synthetic dry air with a CO_2_ concentration of about 1500 µmol mol^−1^ was mixed with CO_2_-free air of controlled humidity to set the CO_2_ concentration of inlet air to 500 µmol mol^−1^. Air flows were controlled with flow controllers. Two of these mixing units were used, one with untreated CO_2_ and the other one with the same CO_2_ that was previously isotopically scrambled at 1000 °C (target Δ_47_ ~ 0.0 ‰, see below for discussion of deviations). The air flow rate was adjusted so that the CO_2_ concentration at the outlet (and thus also in the cuvette) was about 400 µmol mol^−1^. Thus, the decrease in CO_2_ concentration in the cuvette as a result of uptake by the leaf was about 100 µmol mol^−1^. In typical experiments the air flow rate was between 0.6 and 1.5 L min^−1^ depending on the CO_2_ uptake rate of the leaf. It generally took 1 h to reach steady state gas exchange conditions. Gas exchange was measured before and after sampling with an infrared gas analyzer (IRGA) (LI-6262; Li-Cor, Lincoln, NE, USA) operated in the absolute mode. CO_2_ and H_2_O concentrations of inlet and outlet air were measured subsequently. The IRGA used for CO_2_ mole fraction measurements, was calibrated every day with compressed air (dry) which has a known CO_2_ mole fraction, and the reference cell was flushed with CO_2_-free N_2_ gas. For the water mole fraction, the IRGA was calibrated using a dew point mirror, and the reference cell was flushed with CO_2_-free N_2_ gas. Gas exchange variables were calculated according to von Caemmerer and Farquhar ^61^ (see supplementary material Table S1).

The measurements before and after sampling were used to check whether leaves remained sufficiently in steady state over the sampling period, if not, the samples were discarded. The mean value of the two gas exchange measurements (before and after collecting an air sample) were used to calculate the gas exchange parameters. Preparing scrambled CO_2_ is labor intensive, as a result we used normal CO_2_ during the gas exchange experiment until reaching steady state. After the steady state was reached (i.e. constant CO_2_ and H_2_O mole fractions) the CO_2_ source was switched to the scrambled CO_2_ supply (Fig. 4) and we waited 15 min to re-establish steady state conditions. Sampling of air was done by attaching a Mg(ClO_4_)_2_ dryer (Sigma Aldrich, USA) and 6-L of glass flasks to the outlet of the cuvette. The duration of completely flushing and filling the flasks depended on the flow rate and varied between 20 and 50 min.

We regularly determined the isotopic composition of the entering CO_2_ by flushing it through an empty leaf cuvette and sampling the air at the outlet as a blank experiment. We used two different synthetic air cylinders spiked with scrambled CO_2_: The first cylinder had a δ^13^C value of − 2.50 ± 0.02 ‰, a δ^18^O value of 25.3 ± 0.1 ‰ and a Δ_47_ value of 0.24 ± 0.02 ‰; the second cylinder had a δ^13^C value of − 2.43 ± 0.03 ‰, a δ^18^O value of 25.5 ± 0.2 ‰ and a Δ_47_ value of 0.07 ± 0.03 ‰ (Table S1, supplementary material). If not indicated differently, these and all further errors reported in this manuscript are 1 σ standard deviation, determined from repeated analysis of samples.

### Automated CO_2_ extraction from air

**Figure 5:**
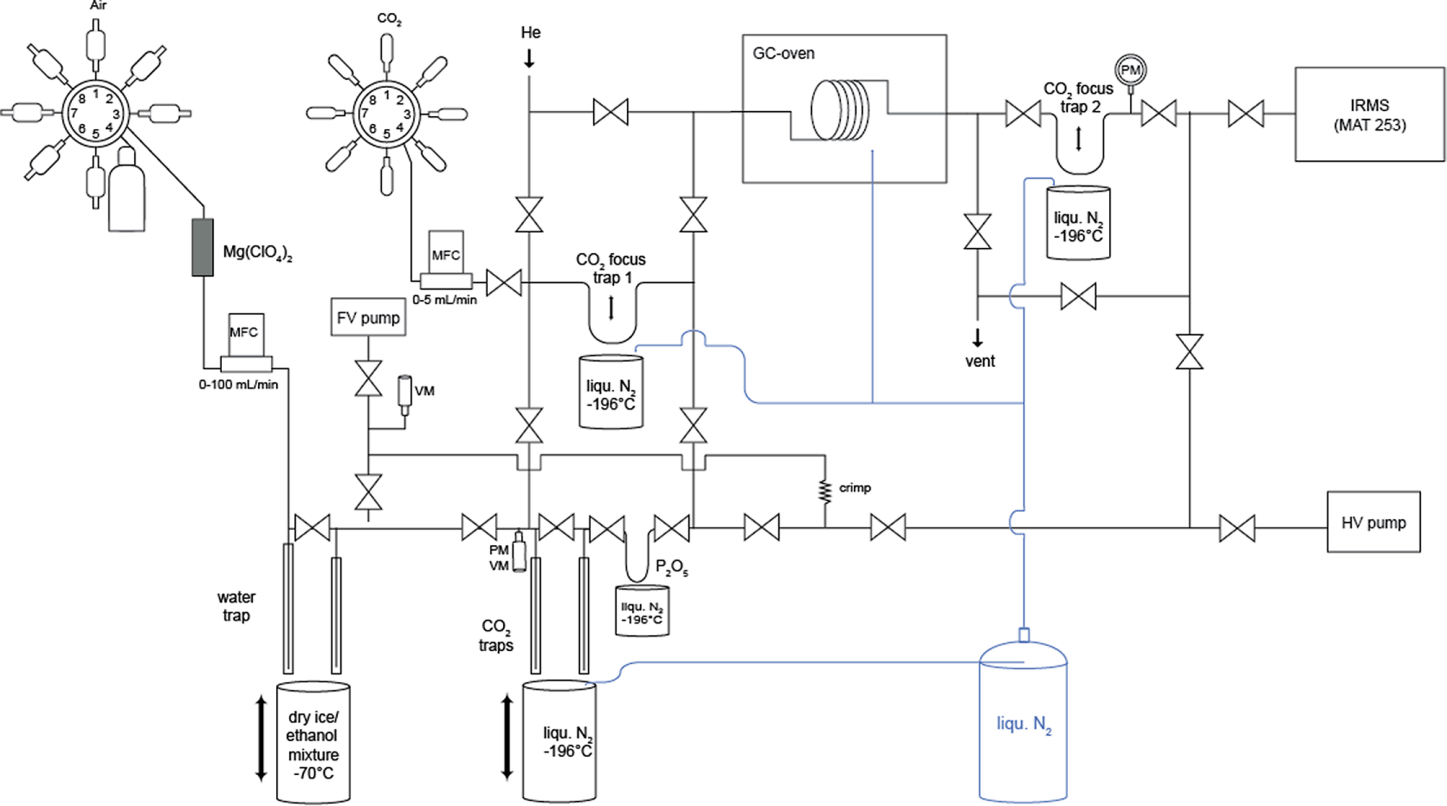
Schematic of the automated system for CO_2_ extraction from air and purification system. The liquid nitrogen tank is connected to the Dewar using Teflon tube covered with a temperature insulator.

We used an automated CO_2_ extraction and purification system to prepare the sample CO_2_ for ^13^C^18^O^16^O analysis. The system was mainly manufactured from stainless steel parts and consisted of four main units: (i) an air inlet system, (ii) chemical and cryogenic CO_2_ drying units, (iii) a cryogenic CO_2_ trap and (iv) a GC column for purification (Fig. 5). The outlet of the extraction line was directly connected to the sample bellows of the mass spectrometer. The design of the air inlet system and the cryogenic water and CO_2_ traps were based on an automated CO_2_ extraction system dedicated to conventional mass spectrometric isotope analysis of atmospheric CO_2_^62^.

The air inlet system allowed connecting several air samples via an 8-port Valco multiport valve: One position of the multiport valve was capped off and was used as a ‘closed’ position, two positions were generally used for working gas cylinders and the remaining five positions could be used to connect the sample flasks. About 2.5 L of air were required for one analysis. For a few samples we measured duplicates. Prior to an extraction sequence, the analytical system was evacuated up to the flask valves. The outlet of the multiport valve was connected to a mass flow controller (10–100 mL min^−1^; MKS Instruments) to regulate the gas flow during the extraction system. The flow rate was routinely set to 50 mL min^−1^ for extracting the CO_2_ from air.

In order to minimize exchange with water the gas was thoroughly dried at three different positions in the automated extraction system: (i) before the mass flow controller with a magnesium perchlorate unit, (ii) after the mass flow controller with two cryogenic water traps, and (iii) after the CO_2_ trap with a phosphorus pentoxide (P_2_O_5_) unit. The magnesium perchlorate was filled in a ca. 10 cm long open-end glass tube with 10 mm inner diameter in the central part and ¼” connectors. The drying agent was held in place with glass wool. The cryogenic water traps were made from 25 cm long stainless steel tubes with a diameter of ¼ ” and inner tubes with a diameter of 1/8 ”^63^. The outer tubes were welded tight at the bottom and electropolished afterwards to reobtain a smooth surface. The inner tubes ended about 0.5 cm above the bottom of the outer tubes to force the air through the entire length of the water trap. To enhance the cold transfer between the outer and inner tube, the inner tubes were slightly bent to touch the outer tubes. The cryogenic water traps were cooled with a mixture of dry ice and ethanol to − 72 °C. The position of the Dewar with the cooling bath was controlled with a hydraulic lift. Due to the relatively large size of the Dewar, the temperature of the cooling bath stayed constant overnight. The (P_2_O_5_) drying unit consisted of a 10 cm long and 6 mm o.d. wide glass finger containing phosphorous pentoxide (P_2_O_5_), which was connected via a 1/8″ stainless steel tube to the CO_2_ trap. It was immersed in liquid nitrogen using a hydraulic lifter.

The chemical drying units were added to the system at a later stage in an attempt to reduce isotopic re-equilibration during the extraction process (see below). However, also these additional measures did not prevent partial re-equilibration in the extraction line so that we had to correct all Δ_47_ data for this partial re-equilibration. The size of the corresponding correction was established by frequently analyzing heated and non-heated CO_2_ in air standards (see Sect. "Isotopic analysis of CO_2_ (δ^13^C, δ^18^O and Δ_47_").

Carbon dioxide (and nitrous oxide) was trapped by immersing the CO_2_ trap in liquid nitrogen. The design of the carbon dioxide trap and cooling unit was the same as the cryogenic water trap described above. During extraction, the air was processed through the system with a fore vacuum pump. A mechanical flow restriction (crimped 1/16″ stainless steel tubing) after the CO_2_ trap slowed down the flow rate in the CO_2_ trap. The crimping was adjusted such that the pressure in the water and CO_2_ trap was about 180 mbar at a flow rate of 50 mL min^−1^.

After trapping the CO_2_ in the liquid nitrogen trap, the trap was warmed up to room temperature and the sample CO_2_ was frozen to the P_2_O_5_ trap. Then, the valve on top of the P_2_O_5_ glass finger was closed and the CO_2_ was brought to room temperature for final drying for about 10 min. Afterwards, the CO_2_ was transferred to a 1/16″ focusing trap in front of the gas chromatographic column, by immersing this focusing trap in liquid nitrogen. The CO_2_ was then released into a helium stream at a flow rate of approx. 25 mL min^−1^ through the gas chromatographic (GC) column packed with Porapak Q. The GC column was cooled to 20 °C during purification with cold nitrogen gas provided from a liquid nitrogen Dewar, to hold back possible hydrocarbon impurities^20^ and it was heated up to 250 °C in-between extractions. After passing through the GC column, the CO_2_ was trapped in a second 1/16″ trap and the helium was evacuated using the fore and high vacuum pump of the isotope ratio mass spectrometer (IRMS). Finally, the pure CO_2_ was warmed to room temperature and injected into the mass spectrometer.

The whole CO_2_ extraction line (including the liquid nitrogen cooling) was controlled via LabVIEW (Version 15.0). The LabVIEW software also gave a signal to the IRMS software Isodat (Version 2.0) to start a measurement. Extraction and purification of one sample took about 4.5 h and we generally analyzed two working standards and three samples per day.

### Isotopic analysis of CO_2_ (δ^13^C, δ^18^O and Δ_47_)

Mass spectrometric analysis of the isotopic composition of the extracted and purified CO_2_ was carried out in dual inlet mode on a MAT 253 mass spectrometer (Thermo Fisher Scientific, Germany) with a modified collector unit that allowed simultaneous analysis of the mass-to-charge ratios (m/z) 44, 45, 46, 47, 48 and 49. The overall reproducibility for Δ_47_ analysis was about 0.04 ‰, determined from replicate measurements of the compressed air cylinder.

The mass spectrometric Δ_47_ measurements were calibrated relative to CO_2_ heated to 1000 °C and CO_2_ equilibrated with water at 28°C^25,64^. We analyzed heated and water equilibrated CO_2_ with a different bulk isotopic composition and determined a slope of 0.0024 for the heated and water-equilibrated gas line (Figure S3, supplementary material). This slope was used to correct for small, negative background effects on m/z 47. The bulk isotopic composition of our in-house reference CO_2_ gas was δ^13^C_VPDB_ = − 2.82 ‰ and δ^18^O_VSMOW_ = − 26.11 ‰ and the maximum δ^47^ difference between the sample and the reference CO_2_ was 17 ‰ resulting in a maximum Δ_47_ bulk isotope composition effect − 0.04 ‰ which is corrected based on heated and equilibration gas slope.

In order to calibrate our δ^13^C, δ^18^O and Δ_47_ measurements of CO_2_ in air, we prepared two cylinders of CO_2_ in synthetic air as working reference cylinders (Table S2). The first synthetic air cylinder was spiked with CO_2_ resulting in a concentration of about 500 µmol mol^−1^, δ^13^C_VPDB_ = − 2.76 ‰, δ^18^O_VSMOW_ = 25.65 ‰ and Δ_47_ =  0.82 ‰. For the second synthetic air cylinder we used scrambled CO_2_ with a similar bulk isotopic composition, i.e. δ^13^C_VPDB_ =  − 2.73 ‰, δ^18^O_VSMOW_ = − 25.83 ‰ but a low clumped isotope signature of Δ_47_ = 0.11 ‰ and a CO_2_ concentration of 400 µmol mol^−1^.

The clumped isotopic composition of the first air cylinder was determined by analyzing the pure CO_2_ directly in the dual inlet system of the IRMS versus the heated and water equilibrated CO_2_ samples and versus CO_2_ obtained by acid digestion at 70 °C from a set of clumped isotope carbonate standards (ETH1, ETH2, ETH3 and ETH4). Both procedures gave consistent results of Δ_47_ = 0.82 ± 0.04 ‰. The same CO_2_ was then mixed in reference air cylinder 1 with synthetic air. After extracting and purifying the CO_2_, we found an average Δ_47_ value of 0.86 ± 0.04 ‰. The reason for this offset of 0.04 ± 0.04 ‰ might be partial re-equilibration within the extraction line, where full equilibration at room temperature would result in Δ_47_ = 0.93 ‰.

For preparing the second air cylinder, we heated the pure CO_2_ to 1000 °C for more than 2 hours^64^ and then mixed it into synthetic, CO_2_-free air. After extracting and purifying the CO_2_ from the second cylinder, we determined an average Δ_47_ value of 0.42 ± 0.04 ‰. The reason for this significant deviation from the expected Δ_47_ value of 0.03 ‰ is most likely twofold: (i) partial re-equilibration during mixing of scrambled CO_2_ with synthetic air, and (ii) partial re-equilibration in the automated CO_2_ extraction line. Subsequently, we prepared several other mixtures of heated CO_2_ with an expected clumped isotope signature of Δ_47_ = 0.03 ‰ in synthetic air cylinders. For all cylinders that were prepared this way we found Δ_47_ values that were at least + 0.26 ‰ enriched in Δ_47_ relative to the expected value. It is not straightforward to decide whether the clumped signal of a CO_2_-in-air standard was altered during the preparation of the air standard or during the CO_2_ extraction step. Comparison between the Δ_47_ of the pure CO_2_ and Δ_47_ value after processing the CO_2_-air mixtures through the CO_2_ extraction line reveals a Δ_47_ scale contraction of 24%, i.e. we measure only 76% of the true difference between samples when determining the clumped isotopic composition of CO_2_ in air. This apparent scale contraction was highly reproducible over the course of the experiments reported here and we corrected all Δ_47_ values accordingly. This illustrates that referencing Δ_47_ measurements for atmospheric CO_2_ samples is challenging because there is no Δ_47_ standard for CO_2_-in-air studies available.

The leaf gas exchange samples contained up to about 0.3 µmol mol^−1^ N_2_O. All CO_2_ isotope measurements of δ^13^C, δ^18^O and Δ_47_ were corrected for the mass interference from the N_2_O isotopologues because N_2_O was not separated from CO_2_ during the purification step. The amount of N_2_O was inferred from the intensity of the N fragment at m/z 14 relative to the intensity of m/z 44^20^. For typical N_2_O mole fractions of 0.3 µmol mol^−1^, the N_2_O correction was approximately + 0.17 ‰ for δ^13^C, + 0.45 ‰ for δ^18^O and − 0.13 ‰ for Δ_47_.

Considering all correction procedures, most notable the 24% Δ_47_ scale contraction, we obtain an overall reproducibility for repeated extraction of CO_2_ from air and subsequent isotopic analysis of 0.08 ‰ for δ^13^C, 0.3 ‰ for δ^18^O and 0.045 ‰ for Δ_47_.

### Leaf water extraction and δ^18^O analysis

Immediately after sampling of air for Δ_47_ measurements, the leaf was placed between plastic sheets, its area was measured, and it was enclosed in a vial and frozen. Leaf water was extracted from the leaves by cryogenic vacuum distillation, i.e. the leaf sample was heated in vacuum to 60 °C and the evaporated water was directly frozen in a vial cooled to liquid nitrogen temperature^13^. The distillation process was carried out for at least 4 h to ensure quantitative extraction (West et al., 2006). The δ^18^O value of the leaf water was determined by equilibrating CO_2_ and water in a GasBench II (Thermo Scientific), and subsequent analysis of the oxygen isotope composition of the equilibrated CO_2_ with a Delta V mass spectrometer (Thermo Scientific, Germany). The oxygen isotope composition was calibrated versus VSMOW and SLAP.

## Results

### Gas exchange data and isotopic composition of leaf water

Gas exchange of *Helianthus* was characterized by high stomatal conductance relative to the other two species (Table 1). The net assimilation *A*_n_ was 12 µmol m^−2^ s^−1^ at a PPFD of 200 µmol m^−2^ s^−1^ and increased to 22 µmol m^−2^ s^−1^ at 700 µmol m^−2^ s^−1^. The net assimilation increased only little further when measured at a PPFD of 1800 µmol m^−2^ s^−1^ (25 µmol m^−2^ s^−1^). *c*_i_/*c*_a_ decreased with increasing irradiance, from 0.82 (at PPFD of 200 µmol m^−2^ s^−1^) to 0.75 (at 1800 µmol m^−2^ s^−1^).

*Hedera* was measured at a PPFD of 700 µmol m^−2^ s^−1^ only where *A*_n_ was lower than that of the other two species (15 µmol m^−2^ s^−1^). Compared to *Helianthus* at the same PPFD, the stomatal conductance *g*_s_ was much lower (0.08 mol m^−2^ s^−1^), causing a clearly lower *c*_i_/*c*_a_ ratio (0.41) (Table 1). The C_4_ species *Zea* was also measured at 700 µmol m^−2^ s^−1^ only where *A*_n_ was the highest of the three (28 µmol m^−2^ s^−1^) but *g*_s_ was rather low (0.13 mol m^−2^ s^−1^) causing the lowest *c*_i_/*c*_a_ ratio of the three species, (0.32) (Table 1).

For *Helianthus*, mesophyll conductance calculated using Δ_A_^13^C (*g*_m13_) increased with light intensity whereas the mesophyll conductance measured using Δ_A_^18^O (*g*_m18_) did not show a clear correlation with the light intensity (Table 1 and Figure S2). Our estimates of *g*_m18_ had relatively larger errors compared to *g*_m13_ (Table 1) and the values were larger (Table 1). For *Helianthus*, the *g*_m18_ estimates were 1.3 to 2.5 times larger and for *Hedera* 1.5 times.

The δ^18^O value of the bulk leaf water of *Helianthus* varied between 4.3 and 6.9 ‰ with an average value of 6 ± 1 ‰. For *Hedera*, the bulk leaf water isotopic composition was δ^18^O = 3.1 ± 1.6 ‰. The relative difference in the δ^18^O value of the bulk leaf water between the *Helianthus* and *Hedera* is due to the difference in the δ^18^O of source water. For *Zea*, the bulk leaf water isotopic composition of the leaf part inserted in the cuvette was δ^18^O = 20.5 ± 1.3 ‰. We used a section of the *Zea* leaves at about 1/3 from the tip for gas exchange experiments and such high enrichments in δ^18^O of leaf water compared to the source water are typical for sections towards the tip of elongated leaves (see, ^65^) and at higher vapor pressure deficit^66^.

### Effect of photosynthetic gas exchange on the isotopic composition of CO_2_

For *Helianthus* Δ_47_ increased from 0.24 ‰ in the incoming air to 0.50 ‰ to 0.61 ‰ in the outgoing air, at a *c*_m_/*c*_a_ ratio of 0.66 to 0.78 (Table 1 and 2). For *Hedera*, at a *c*_m_/*c*_a_ ratio of 0.28, the change in Δ_47_ between incoming and outgoing air was more variable than for the other species at similar light intensity. The average change of all the experiments under similar conditions is insignificant (from 0.24 ‰ to 0.22 ‰). For *Zea*, at lower *c*_m_/*c*_a_ ratio (0.20), results were more consistent and no statistically significant decrease in Δ_47_ between incoming and outgoing air was observed (Table 1 and 2).

**Table 2:** Isotopic composition of the CO_2_ entering and leaving the leaf cuvette and the resulting isotopic discrimination against the isotopologues ^13^C^16^O^16^O, ^12^C^18^O^16^O and ^13^C^18^O^16^O given as Δ_A_^13^C, Δ_A_^18^O and Δ_A_Δ_47_. All the isotope and discrimination values are reported in per mill (‰), with respect to VPDB (for δ^13^C) and VSMOW (for δ^18^O). The values in bold are mean and standard deviation for the replicates at different light conditions.

Species	PPFD	No. exp	Cyl	δ^13^C_e_	δ^13^C_a_	Δ_A_^13^C	δ^18^O_e_	δ^18^O_a_	Δ_A_^18^O	Δ_47e_	Δ_47a_	Δ_A_Δ_47_
*H. annuus*												
	200	(1)	I	− 2.50 ± 0.02	0.99 ± 0.06	22.5 ± 0.3	25.3 ± 0.1	36.2 ± 0.3	71 ± 2	0.24 ± 0.02	0.54 ± 0.05	1.9 ± 0.3
		(2)	I	− 2.50 ± 0.02	1.20 ± 0.06	22.5 ± 2.3	25.3 ± 0.1	39.2 ± 0.3	77 ± 2	0.24 ± 0.02	0.61 ± 0.05	2.0 ± 0.3
		(3)	I	− 2.50 ± 0.02	1.73 ± 0.06	23.5 ± 0.3	25.3 ± 0.1	40.0 ± 0.3	84 ± 2	0.24 ± 0.02	0.68 ± 0.05	2.4 ± 0.3
	**Mean ± SD**		**− 2.50**	**1.31 ± 0.38**	**22.8 ± 0.6**	**25.3**	**38.5 ± 2**	**77.3 ± 7**	**0.24**	**0.61 ± 0.07**	**2.10 ± 0.26**	
	700	(4)	I	− 2.50 ± 0.02	1.78 ± 0.06	21.6 ± 0.3	25.3 ± 0.1	36.3 ± 0.3	56 ± 2	0.24 ± 0.02	0.48 ± 0.05	1.2 ± 0.3
		(5)	I	− 2.50 ± 0.02	1.22 ± 0.06	20.5 ± 0.3	25.3 ± 0.1	34.8 ± 0.3	52 ± 2	0.24 ± 0.02	0.58 ± 0.05	1.9 ± 0.3
		(6)	I	− 2.50 ± 0.02	1.51 ± 0.06	21.3 ± 0.3	25.3 ± 0.1	35.8 ± 0.3	56 ± 2	0.24 ± 0.02	0.57 ± 0.05	1.7 ± 0.3
		(7)	I	− 2.50 ± 0.02	1.65 ± 0.06	22.5 ± 0.3	25.3 ± 0.1	36.8 ± 0.3	63 ± 2	0.24 ± 0.02	0.61 ± 0.05	2.0 ± 0.3
	**Mean ± SD**			**− 2.50**	**1.54 ± 0.24**	**21.5 ± 0.8**	**25.3**	**35.9 ± 0.9**	**56.8 ± 5**	**0.24**	**0.56 ± 0.06**	**1.7 ± 0.36**
				− 2.50	1.54 ± 0.24	21.5 ± 0.8	25.3	35.9 ± 0.9	56.8 ± 5	0.24	0.56 ± 0.06	1.70 ± 0.36
	1800	(8)	I	− 2.50 ± 0.02	1.34 ± 0.06	19.8 ± 0.3	25.3 ± 0.1	35.6 ± 0.3	53 ± 2	0.24 ± 0.02	0.45 ± 0.05	1.1 ± 0.3
		(9)	I	− 2.50 ± 0.02	1.18 ± 0.06	20.4 ± 0.3	25.3 ± 0.1	34.6 ± 0.3	53 ± 2	0.24 ± 0.02	0.50 ± 0.05	1.3 ± 0.3
		(10)	I	− 2.50 ± 0.02	1.52 ± 0.06	21.3 ± 0.3	25.3 ± 0.1	35.8 ± 0.3	56 ± 2	0.24 ± 0.02	0.55 ± 0.05	1.6 ± 0.3
	**Mean ± SD**		**− 2.50**	**1.35 ± 0.17**	**20.5 ± 0.8**	**25.3**	**35.3 ± 0.6**	**54 ± 2**	**0.24**	**0.50 ± 0.05**	**1.33 ± 0.25**	
***H.hibernica***	700	(1)	I	− 2.50 ± 0.02	− 0.53 ± 0.06	10.5 ± 0.3	25.3 ± 0.1	28.8 ± 0.3	18 ± 2	0.24 ± 0.02	0.25 ± 0.05	0.0 ± 0.3
		(2)	I	− 2.50 ± 0.02	− 0.64 ± 0.06	9.4 ± 0.3	25.3 ± 0.1	28.4 ± 0.3	15 ± 2	0.24 ± 0.02	0.29 ± 0.05	0.3 ± 0.3
		(3)	I	− 2.50 ± 0.02	− 0.35 ± 0.06	10.5 ± 0.3	25.3 ± 0.1	29.2 ± 0.3	19 ± 2	0.24 ± 0.02	0.12 ± 0.05	− 0.5 ± 0.3
	**Mean ± SD**			**− 0.51 ± 0.15**	**10.13 ± 0.6**	**25.3**	**28.80 ± 0.4**	**17.33 ± 2**	**0.24**	**0.22 ± 0.09**	**− 0.07 ± 0.4**	
***Z. mays***	700	(1)	II	− 2.43 ± 0.03	− 1.75 ± 0.06	3.5 ± 0.3	25.5 ± 0.2	28.4 ± 0.3	15 ± 2	0.07 ± 0.03	0.07 ± 0.05	0.0 ± 0.3
		(2)	II	− 2.43 ± 0.03	− 1.75 ± 0.06	3.9 ± 0.3	25.5 ± 0.2	28.3 ± 0.3	16 ± 2	0.07 ± 0.03	0.05 ± 0.05	− 0.1 ± 0.3
		(3)	II	− 2.43 ± 0.03	− 1.78 ± 0.06	3.3 ± 0.3	25.5 ± 0.2	31.6 ± 0.3	31 ± 2	0.07 ± 0.03	0.05 ± 0.05	− 0.1 ± 0.3
	**Mean ± SD**		**− 2.43**	**− 1.76 ± 0.02**	**3.57 ± 0.31**	**25.5**	**29.43 ± 1.9**	**20.67 ± 9**	**0.07**	**0.06 ± 0.01**	**− 0.07 ± 0.06**	

**Figure 6:**
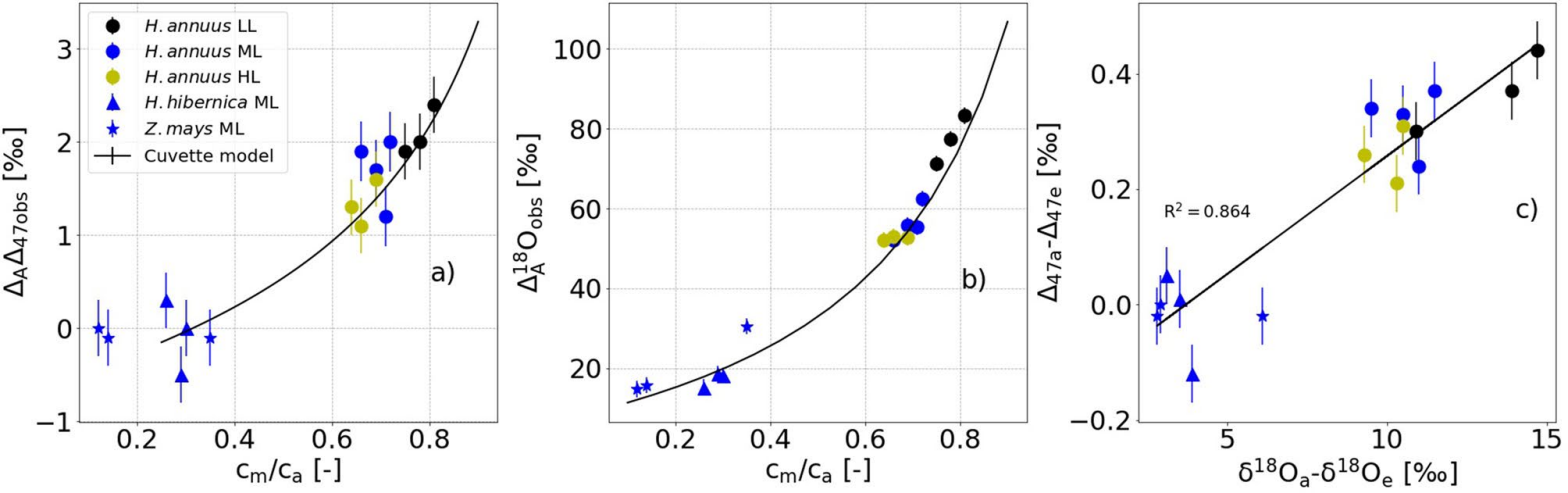
Effect of photosynthetic gas exchange on Δ_47_ and δ^18^O of CO_2_. Δ_A_Δ_47_ (**a**) and Δ_A_^18^O (**b**) during photosynthetic gas exchange experiments as a function of the *c*_m_/*c*_a_ ratio. (LL = low light: PPFD = 200 μmol m^− 2^ s^−1^, ML = medium light: PPFD = 700 μmol m^−2^ s^−1^, HL = high light: PPFD = 1800 μmol m^−2^ s^−1^). a) discrimination against ^13^C^18^O^16^O (Δ_A_Δ_47_). (**b**) discrimination against ^12^C^18^O^16^O (Δ_A_^18^O). (**c**) Relative difference between Δ_47_ the CO_2_ entering and leaving the cuvette as a function of the difference between δ^18^O of CO_2_ entering and leaving the cuvette. The solid line is a linear regression fit with a function of (Δ_47a_− Δ_47a_) = (0.041 ± 0.004) × (δ^18^Oa–δ^18^Oe)—0.151 ± 0.040. For the leaf cuvette model, we assumed δ^18^O = 10 ‰ for the leaf water and a mesophyll conductance of 0.5 mol m^−2^ s^−1^ bar^−1^.

When these changes are converted to discrimination (Δ_A_Δ_47_), for *Helianthus*, we observed an average Δ_A_Δ_47_ of 1.7 ± 0.4 ‰. Slightly negative but non-significant discriminations were observed for *Hedera* (− 0.07 ± 0.4 ‰), and *Zea* (− 0.07 ± 0.06 ‰) (Fig. 6a, Table 2). The Δ_A_Δ_47_ correlates strongly with the *c*_m_/*c*_a_ ratio, higher Δ_A_Δ_47_ are observed at higher *c*_m_/*c*_a_ ratio. The change in Δ_47_ between CO_2_ entering and leaving the cuvette correlates strongly with the change in δ^18^O of CO_2_ entering and leaving the cuvette, with an R^2^ value of 0.864 (Fig. 6c). The positive correlation between Δ_47_ and δ^18^O of CO_2_ indicates that photosynthetic gas exchange affects Δ_47_ and δ^18^O similarly (Fig. 6a).

In addition to the Δ_A_Δ_47_, we also measured Δ_A_^13^C and Δ_A_^18^O (Table 2). The average net carbon isotope discrimination was Δ_A_^13^C = 21.6 ± 1.2 ‰ for *Helianthus*, 10.1 ± 0.7 ‰ for *Hedera* and 3.6 ± 0.3 ‰ for *Zea*. The magnitude of Δ_A_^13^C also correlates with the *c*_c_/*c*_a_ ratio, in agreement with previous studies^13,42,48^. As *c*_c_/*c*_a_ depends on light intensity, the gas exchange experiments with *Helianthus* show a slightly higher Δ_A_^13^C at low light intensity (22.8 ± 0.6 ‰) compared to mid and high light conditions (21.1 ± 0.9 ‰). The average apparent oxygen isotope discrimination Δ_A_^18^O was 62 ± 12 ‰ for *Helianthus*, 18 ± 3 ‰ for *Hedera* and 21 ± 9 ‰ for *Zea*. Similar to Δ_A_^13^C, Δ_A_^18^O is higher at low light intensities (78 ± 6 ‰) compared to mid and high light conditions (55 ± 4 ‰) (Table 2, Fig. 6b).

## Discussion

The Δ_47_ value of CO_2_ has been suggested as a possible tracer for gross primary production, however two previous studies presented contradicting conclusions on the effect of photosynthesis on the Δ_47_ value of CO_2_^19,22^. In this study, using a leaf cuvette experiment under controlled conditions (light, CO_2_, temperature and humidity) and a leaf cuvette model, we showed that photosynthetic gas exchange can in principle increase or decrease the Δ_47_ value of CO_2_ depending on the Δ_47_ value of the CO_2_ entering the leaf, the CO_2_–H_2_O exchange temperature and the back-diffusion flux (quantified as *c*_m_/*c*_a_ ratio). However, under conditions similar for the current atmosphere, photosynthesis depletes the Δ_47_ value of atmospheric CO_2_.

**Figure 7:**
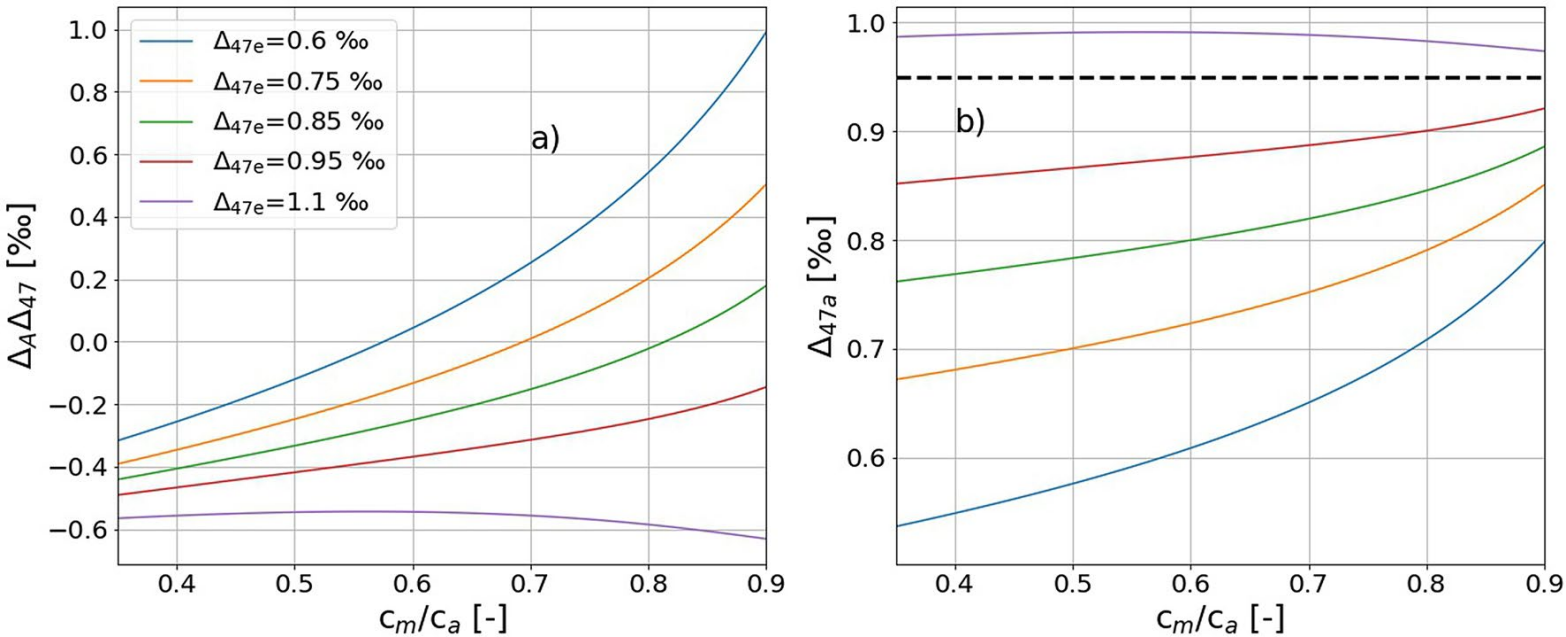
Leaf cuvette model result for Δ_47_ photosynthetic discrimination. (**a**) Δ_A_Δ_47_ as a function of *c*_m_/*c*_a_ for various value of Δ_47e_ (see legend). Panel (**b**) shows the corresponding value for Δ_47a_, the black dashed line indicates the Δ_47_ of CO_2_ at the CO_2_-H_2_O exchange site for the leaf temperature of 20 °C.

The photosynthetic effect on Δ_47_ of the residual CO_2_ for the C_3_ species *Helianthus* and *Hedera* correlated with the CO_2_ concentration gradient over the leaf, *i.e. c*_m_/*c*_a_, and the discrimination in Δ_47_ showed a similar pattern to Δ_A_^18^O (Fig. 6). The main driver for the discrimination against δ^18^O and Δ_47_ values of CO_2_ is isotope exchange with leaf water, and the fractionation associated with the initial fixation by the enzyme RuBisCO (Ribulose-1,5-bisphosphate carboxylase-oxygenase) or PEP (Phosphoenolpyruvate) has no/negligible effect on the δ^18^O and Δ_47_ value of CO_2_. Δ_47_ value is independent of fractionations in the bulk isotope composition (i.e., variations in δ^18^O value due to isotope exchange with leaf water and changes in δ^13^C due to metabolic carbon fixation). The lower discrimination in Δ_47_ by C_4_ plant *Zea* is due to the lower back-diffusion flux (lower conductance and higher assimilation rate) and the fractionation is dominated by diffusion in agreement with the hypothesis of Eiler and Schauble^19^. Gas phase diffusion causes a decrease in Δ_47_ of the residual CO_2_^19^, see also Fig. 7.

For *Helianthus*, at a *c*_m_/*c*_a_ ratio of 0.81, we observed an increase in Δ_47_ from 0.24 ‰ to 0.68 ‰ (an increase by 0. 44 ‰) for a CO_2_ drawdown of 100 µmol mol^−1^. Eiler and Schauble^19^ reported an increase in Δ_47_ from 0.65 to 0.75 (an increase by 0.1 ‰, i.e. Δ_A_ Δ_47_ ~ 0.54 ‰) due to photosynthetic gas exchange for a CO_2_ drawdown of 72 µmol mol^−1^ (from 390 to 318) using *Antirrhinum majus* with a *c*_m_/*c*_a_ ratio of 0.85, which is consistent with our results.

Using the leaf cuvette model, we quantitatively estimated the expected Δ_47_ discrimination based on the equations discussed in Sect. “Photosynthetic Δ_47_ discrimination" and the derived concentration gradient between the atmosphere and the site of CO_2_–H_2_O exchange (*c*_m_/*c*_a_). For the C_3_ species, we obtained an excellent agreement between the observed and the predicted change in Δ_47_, except for one outlier for the experiments with *Hedera* (Fig. 6 and Figure S5). For *Zea*, the observed change between outgoing and ingoing CO_2_ of − 0.01 ± 0.01 ‰ (the error is standard deviation for the three measurements) was slightly lower than the expected change in Δ_47_ of about + 0.01 ‰. However, the difference was still of the order of our measurement precession.

The correlation between Δ_A_Δ_47_ and the *c*_m_/*c*_a_ ratio and the overall good agreement between observed and predicted Δ_A_Δ_47_ confirm the hypothesis of Eiler and Schauble^19^ that CO_2_–H_2_O exchange and kinetic fractionation control the discrimination. For high CO_2_ back-diffusion fluxes, *i.e.,* high *c*_m_/*c*_a_ ratios, the magnitude of the in- and outgoing CO_2_ flux is almost the same so that the kinetic term due to diffusion cancels out and the Δ_47_ value of the residual CO_2_ is close to the thermodynamic equilibrium value for the respective leaf temperature, *i.e.* Δ_47_ = 0.95 ‰ at 20 °C (Fig. 2). At room temperature, the sensitivity of Δ_47_ to temperature is about 0.005 ‰/°C so that small variations in temperature do not have a large effect. For *c*_m_/*c*_a_ ratios close to zero, the back-diffusion flux is small and the kinetic fractionation term due to diffusion into the intercellular airspace induces a negative discrimination in Δ_47_ of up to − 0.08 ‰ if the ingoing CO_2_ has a Δ_47_ close to 0 ‰.

As mentioned above and illustrated in Fig. 3, we used CO_2_ with an artificially diminished Δ_47_ value in the gas exchange experiments in order to increase the signal, i.e., the Δ_47_ difference between incoming and outgoing CO_2_. After having verified the Eiler and Schauble^19^ mechanism, we use our leaf cuvette model to quantify the effect of photosynthesis on Δ_A_Δ_47_ for CO_2_ with typical ambient Δ_47_ values. In ambient air, Δ_47_ of CO_2_ is usually close to or lower than the Δ_47_ value of CO_2_ at the CO_2_–H_2_O exchange site (Δ_47_ = 0.95 ‰).

Figure 7 illustrates the calculated dependence of Δ_A_Δ_47,_ as well as Δ_47a_ on *c*_m_/*c*_a_ during gas exchange for Δ_47e_ values between 0.6 ‰ and 1.1 ‰ to get Δ_47a_ values between 0.6 ‰ and 1.05 ‰ close to the Δ_47_ values of atmospheric CO_2_ reported in literature^19–22^. A negative Δ_A_Δ_47_ means that $_{{47{\text{a}}}} < _{{47{\text{e}}}}$ (Eq. 9), i.e., photosynthetic gas exchange would decrease ambient Δ_47_ values, whereas a positive Δ_A_Δ_47_ would increase ambient Δ_47_ values. Figure 7a shows that Δ_A_Δ_47_ is mostly negative, thus photosynthetic gas exchange generally acts to decrease Δ_47_ except for situations in which ambient Δ_47_ is at the low end of reported values and *c*_m_/*c*_a_ ratios are very high (indicating very slow assimilation rates).

At very low *c*_m_/*c*_a_ ratio, i.e., the diffusion limited case, Δ_A_Δ_47_ is controlled by the diffusional fractionation. Figure 7a shows that Δ_A_Δ_47_ converges to − 0.5 ‰ as *c*_m_/*c*_a_ approaches 0, independent of the incoming CO_2_. As indicated in Fig. 2, the theoretically calculated Δ_47_ fractionation associated with diffusion is Δ_47a_–Δ_47e_ = − 0.3 ‰ when all CO_2_ that enters the stomata is assimilated. In our leaf cuvette model where we assume a CO_2_ drawdown from 500 to 400 ppm (ζ = 500 / (500–400) = 5), this translates to Δ_A_Δ_47_ ≈ ζ × (Δ_47a—_Δ_47e_) =− 1.5 ‰). The fact that the model assumes that 2/3 of the CO_2_ leave the stomata again without exchanging isotopes explains quantitatively why the diffusion limited endmember is ζ × (Δ_47a—_Δ_47e_)/3 = − 0.5 ‰, independent of the Δ_47_ of CO_2_ entering the cuvette. Figure 7a shows that in this limit the value of Δ_47a_ depends very strongly on Δ_47e_.

For the other extreme scenario *c*_m_/*c*_a_ ≈ 1, Δ_47a_ converges to the Δ_47_ value of CO_2_ at the CO_2_–H_2_O exchange site (Δ_47_ = 0.95 ‰), independent of the Δ_47_ value of incoming CO_2_. This reflects the exchange dominated case in Fig. 2. In this case, Δ_A_Δ_47_ strongly depends on Δ_47e_ value of the CO_2_, for instance Δ_A_Δ_47_ will be ≈ 0.00 ‰ and ≈ − 0.75 ‰ for Δ_47e_ of 0.95 ‰ and 1.1 ‰, respectively. This is similar to what Adnew et al.^13^ showed for the ^17^O-excess of CO_2_ in gas exchange experiments.

The leaf cuvette model calculations show that in principle photosynthetic gas exchange can deplete or enrich the Δ_47_ depending on the initial Δ_47_ value of the CO_2_ in the air surrounding the leaf, leaf temperature (via the Δ_47_ value at the exchange site) and the fraction of CO_2_ exchanged and diffused back to the atmosphere. However, photosynthesis will enrich Δ_47_ only if the Δ_47_ value of the CO_2_ entering the leaf is far lower than the Δ_47_ value of the CO_2_ at the CO_2_–H_2_O exchange site. In addition, Fig. 7b shows that when $\Delta _{{47{\text{e}}}}$ is lower than the equilibrium value of 0.95 ‰, photosynthetic gas exchange cannot lead to $\Delta _{{47{\text{a}}}}$ values above the equilibrium value. Thus, according to our leaf cuvette model, photosynthesis cannot lead to Δ_47_ values that are higher than the thermodynamic equilibrium (unless the incoming, i.e. ambient Δ_47_ values are already higher, in which case they would be reduced). This agrees well with most reported Δ_47_ values of atmospheric CO_2_ which are lower than expected from the CO_2_–H_2_O equilibrium at the surface temperature^19–21^.

As a result, an enrichment of up to 0.08 ‰ in Δ_47_ relative to the thermodynamic equilibrium value reported by Laskar and Liang^22^ for Δ_47_ measurements of CO_2_ sampled in a greenhouse cannot be explained by photosynthesis based on our leaf cuvette model results, even at the extreme scenario of *c*_m_/*c*_a_ ~ 1. In addition, our results show that photosynthetic driven CO_2_–H_2_O isotope exchange affects Δ_47_ and δ^18^O in a similar way (Fig. 6, Figure S3 and S4) as observed for a simple CO_2_–H_2_O equilibration experiment^32,33^. This does not support the greenhouse experiment results of Laskar and Liang^22^ where they concluded that photosynthesis decouples δ^18^O and Δ_47_. We suggest that other processes than photosynthetic gas exchange affected the greenhouse gas experiments reported earlier^22^. Further experiments in similar environments should be carried out to investigate this in more detail.

Our results provide the experimental verification of the isotope exchange model suggested by Eiler and Schauble^19^. In particular, we determine how Δ_A_Δ_47_ varies as a function of *c*_m_*/c*_a_. At high *c*_m_/*c*_a_ ratio (high back-diffusion flux), the effect of fractionation due to diffusion is negligible and the Δ_47_ of atmospheric CO_2_ will be driven towards the Δ_47_ of CO_2_ at the CO_2_–H_2_O exchange site. At low *c*_m_*/c*_a_ ratio, the diffusion fractionation dominates and photosynthetic gas exchange will generally lower Δ_47_. For the real atmosphere with Δ_47_ values slightly lower than the thermodynamic equilibrium set by CO_2_–H_2_O exchange, photosynthetic gas exchange cannot increase Δ_47_ above this equilibrium value.

Our results also show that Δ_47_ and ^18^O discrimination are affected in similar ways during photosynthesis, but in contrast to ^18^O, the clumped isotope composition is independent of the δ^18^O of bulk leaf water. This means that it is not necessary to know the precise isotopic composition of water at the CO_2_–H_2_O exchange site for calculating Δ_A_Δ_47_. Furthermore, a disequilibrium in Δ_47_ is often identified more readily than in δ^18^O, since Δ_47_ depends mainly on CO_2_–H_2_O exchange temperature. As a result, measurements of Δ_47_ during air-leaf gas exchange experiments may be an alternative method to determine the mesophyll conductance to the site of CO_2_–H_2_O exchange and/or the degree of equilibration between CO_2_–H_2_O inside the leaf. The limitation to this approach is that the Δ_47_ signals are very small and it requires high precision measurements to constrain the relevant parameters significantly under ambient conditions.

For *Helianthus*, we found *g*_m13_ values of 0.27 ± 0.1 mol m^−2^ s^−1^ bar^−1^ at a PPFD of 200 μmol m^−2^ s^−1^_,_ 0.54 ± 0.1 mol m^−2^ s^−1^ bar^−1^ at a PPFD of 700 μmol m^−2^ s^−1^ and 0.43 ± 0.05 mol m^−2^ s^−1^ bar^−1^ at a PPFD of 1800 μmol m^−2^ s^−1^, in good agreement with values reported in previous studies^41,43,67,68^. These observations confirm earlier findings that the mesophyll conductance is generally lower at low light intensities (Flexas et al., 2007), although we did not observe any significant difference between mid and high light conditions. For *Hedera*, we found a *g*_m13_ value of 0.20 ± 0.02 mol m^−2^ s^−1^ bar^−1^ at a PPFD of 700 μmol m^−2^ s^−1^, which is in good agreement with the maximum mesophyll conductance of 0.14 ± 0.01 mol m^−2^ s^−1^ bar^−1^ for evergreen angiosperms, including observations from the *Hedera* species^41,43,51,69^. The higher mesophyll conductance for *Helianthus* compared to *Hedera* might be due to the high mesophyll porosity and thin cell walls of mesophyll cells which facilitate easier movement of CO_2_ within intercellular airspaces and across cell walls as reported for evergreen woody plants^70,71^.

Mesophyll conductance (*g*_*m*18_) of *Helianthus* did not show a clear dependency on PPFD (Table 1 and Figure S2) with on average a value of 0.68 mol m^−2^ s^−1^ bar^−1^, which is in good agreement with the few values reported in the literature^41,72^. For maize, *g*_m18_ = 0.57 mol m^−2^ s^−1^ bar^−1^, which is within the wide range of 0.169 to 0.9 mol m^−2^ s^−1^ bar^−1^ reported in literature^41,73–75^. For *Helianthus* and *Hedera*, *g*_*m*18_ is on average 1.7 times *g*_*m*13_ confirming that CO_2_–H_2_O exchange occurs in the mesophyll cell, i.e., before the carboxylation site, in agreement with previous findings^41,47,49,54^.

## Supplementary Information

Supplementary files.

## Data Availability

All the data used in this manuscript are presented in the form of Figures and Tables**.**
