# Supplementary material for: Leaf scale quantification of the effect of photosynthetic gas exchange on Δ47of CO2
Source: Sci Rep. 2021 Jul 7;11:14023. doi: 10.1038/s41598-021-93092-0 (PMC8263724; doi:10.1038/s41598-021-93092-0)
Supplement: Supplementary file 1 — Supplementary files. [file 41598_2021_93092_MOESM1_ESM.pdf]

Supporting Information

Leaf scale quantification of the effect of photosynthetic gas exchange on Δ_{47} of CO_2

Getachew Agmuas Adnew[1*^], Magdalena E.G. Hofmann[1^+], Thijs L. Pons[2], Gerbrand Koren[3], Martin Ziegler[4], Lucas J. Lourens[4], Thomas Röckmann[1]

[1] Institute for Marine and Atmospheric research Utrecht, Utrecht University, Utrecht, The Netherlands

[2] Institute of Environmental Biology, Utrecht University, Utrecht, The Netherlands

[3] Meteorology and Air Quality Group, Wageningen University, Wageningen, The Netherlands

[4] Department of Earth Sciences, Utrecht University, Utrecht, The Netherlands

^Both authors contributed equally

*Corresponding author (g.a.adnew@uu.nl)

[+]now at Picarro B.V., 's-Hertogenbosch, The Netherlands

Mesophyll conductance

Mesophyll conductance to the site of carboxylation, chloroplast (for C_3 plants only)

The degree of the observed discrimination against $^{13}CO_2$ can be calculated theoretically following Busch, et al. [1] as:

$$\Delta_A{}^{13}C_{obs} = \left(\frac{1}{1-t}\right)\left[a_{13b}\frac{c_a-c_s}{c_a} + a_{13s}\frac{c_s-c_i}{c_a}\right] + \left(\frac{1+t}{1-t}\right)\left[a_m\frac{c_i-c_c}{c_a} + b\frac{c_c}{c_a} - \frac{\alpha_b}{\alpha_e\alpha_R}e'\frac{R_D}{A}\frac{c_c}{c_a} - \frac{\alpha_b}{\alpha_f\alpha_R}f\frac{\Gamma^*}{c_a}\right] \quad \text{S1}$$

The expected $\Delta_A{}^{13}C$ ($\Delta_A{}^{13}C_i$) for a scenario of infinite g_m ($c_c = c_i$) is described as:

$$\Delta_A{}^{13}C_i = \left(\frac{1}{1-t^{13}}\right)\left[a_{13b}\frac{c_a-c_s}{c_a} + a_{13s}\frac{c_s-c_i}{c_a}\right] + \left(\frac{1+t^{13}}{1-t^{13}}\right)\left[b\frac{c_c}{c_a} - \frac{\alpha_b}{\alpha_e\alpha_R}e'\frac{R_D}{A}\frac{c_c}{c_a} - \frac{\alpha_b}{\alpha_f\alpha_R}f\frac{\Gamma^*}{c_a}\right] \quad \text{S2}$$

g_{m13} can then be calculated from the difference $\Delta_A{}^{13}C_i$ - $\Delta_A{}^{13}C_{obs}$ (equation S3). The detailed derivation of the equations is extensively described by [1,2]. The estimate of g_m obtained from $\Delta_A{}^{13}C$ is referred to as g_{m13}.

$$g_{m13} = \frac{A_n/P}{c_i - c_c} = \left(\frac{1 + t^{13}}{1 - t^{13}}\right)\left(\frac{A_n\left(b - a_m - \frac{\alpha_b}{\alpha_e} e' \frac{R_D}{R_D + A}\right)}{\left(\Delta_A{}^{13}C_i - \Delta_A{}^{13}C_{obs}\right)Pc_a}\right) \tag{S3}$$

Mesophyll conductance to the CO_2-H_2O exchange site (for both C_3 and C_4 plants)

Gillon and Yakir [3] suggested that the discrimination against ^{18}O of CO_2 during gas exchange can be used to determine the CO_2 conductance from the intercellular air space to the site of CO_2-H_2O equilibration for both C_3 and C_4 plants. The mesophyll conductance from the intercellular air space to the CO_2-H_2O equilibration site can be calculated as shown in equation S6 following [2,4-6] under the assumption that the degree of equilibration between CO_2 and H_2O is 100%.

$$\delta^{18}O_m = \delta^{18}O_A\left(1 - \frac{c_i}{c_m}\right)\alpha^{18}{}_w + \frac{c_i}{c_m}\left(\delta^{18}O_i - a^{18}{}_w\right) + a^{18}{}_w \tag{S4}$$

The CO_2 mole fraction at the site of CO_2-H_2O exchange can be calculated from $\delta^{18}O$ of CO_2 following [2,4,5,7] as shown in equation S5 by rearranging equation S4:

$$c_m = c_i\left(\frac{\delta^{18}O_i - a_{18w} - \delta^{18}O_A(1 + a_{18w})}{\delta^{18}O_m - a_{18w} - \delta^{18}O_A(1 + a_{18w})}\right) \tag{S5}$$

$$g_{m18} = \frac{A_n/P}{c_i - c_m} \tag{S6}$$

After substituting equation S5 into equation S6 and rearrangement, g_{m18} can be expressed as:

$$g_{m18} = \frac{A_n/P}{c_i - c_m} = \frac{A_n/P}{c_i}\frac{\delta^{18}O_A\alpha_{18w} + a_{18w} - \delta^{18}O_m}{\delta^{18}O_i - \delta^{18}O_m} \tag{S7}$$

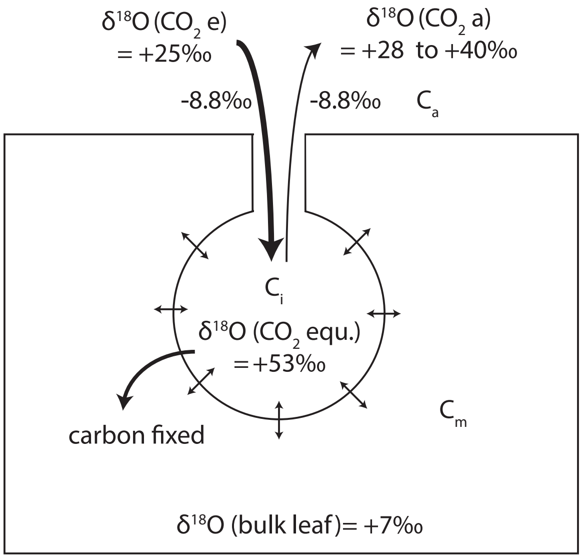

Figure S1: Schematic illustration of the [18]O discrimination of CO_2 during air-leaf gas exchange. The [18]O discrimination is mainly controlled by CO_2-water exchange in the mesophyll and kinetic fractionation during diffusion into and out of the stomata.

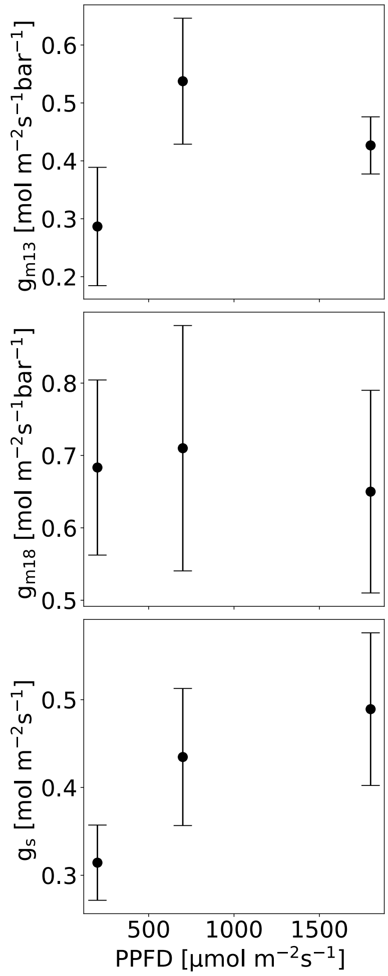

Figure S2: Dependence of mesophyll conductance (a) g_{m13} and b) g_{m18}) and stomatal conductance (c) on PPFD. Error bars denote the 1 σ standard deviation of the mean.

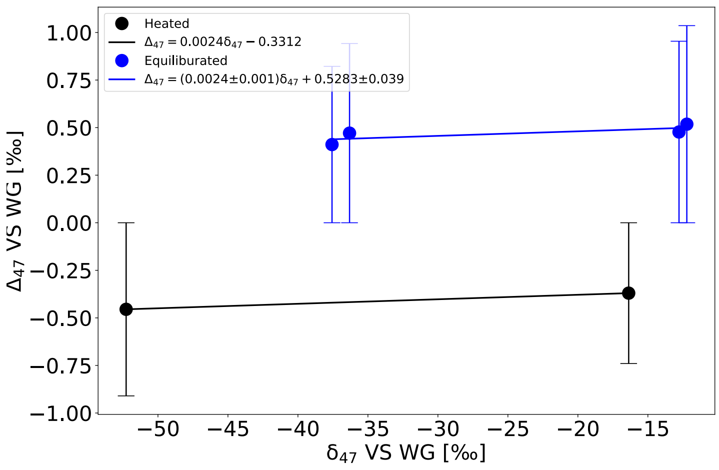

Figure S3: Least-squared linear regression lines for the two CO_2 gases with a different bulk isotopic composition (δ^{47}) heated to 1000°C (black symbols) and equilibrated with water at 28°C (blue symbols).

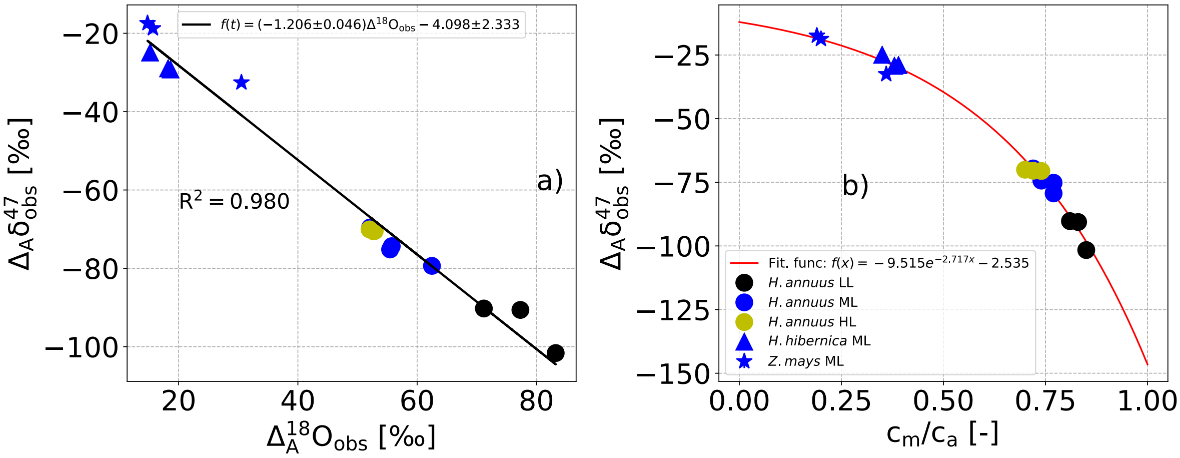

Figure S4: a) Dependence of discrimination against $^{13}C^{16}O^{18}O$ on the discrimination against $^{12}C^{16}O$ ^{18}O. b) Dependence of discrimination in δ^{47} ($\Delta_A\delta^{47}$, see for the equation in Table S1) on the c_m/c_a ratio.

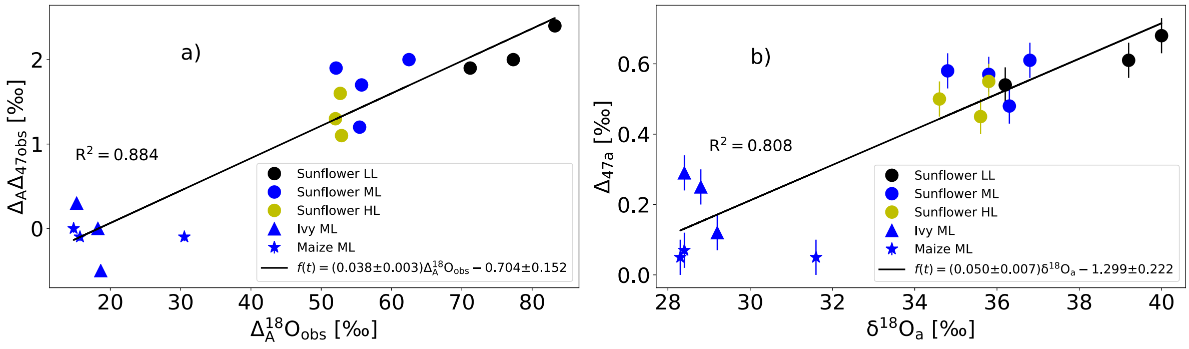

Figure S5: a) Dependence of discrimination against Δ_{47} on the discrimination against ^{18}O. b) Dependence of Δ_{47} of CO_2 leaving the leaf cuvette on the δ^{47} on the $\delta^{18}O$ value of the CO_2 leaving the cuvette.

Table S1 List of variables and equations used in this study to calculate gas exchange parameters and carbon and oxygen isotope discrimination

Symbol	Description	Unit/calculation/value
**Gas exchange**		
A_n	Rate of CO_2 assimilation	$\frac{u_e}{s}\left(c_e - c_a\left(\frac{1-w_e}{1-w_a}\right)\right)$, mol m^{-2}s^{-2}
E	Transpiration rate	$\frac{u_e}{s}\left(\frac{w_a-w_e}{1-w_a}\right)$, mol m^{-2}s^{-2}
w_i	Mole fraction of water vapour inside a leaf	$\frac{613.65\times e^{\left(\frac{17.502\times T_{leaf}}{240.97+T_{leaf}}\right)}\times 10^{-5}}{P}$, mol mol^{-1}
w_a	Mole fraction of water vapour leaving the cuvette /leaf surrounding	mol mol^{-1}
w_e	Mole fraction of water vapour entering the cuvette	mol mol^{-1}
c_e	Mole fraction of CO_2 entering the cuvette	mol mol^{-1}
c_a	Mole fraction of CO_2 in the leaf surrounding/ leaving the cuvette	mol mol^{-1}
u_e	Flow rate of air entering the cuvette	mol s^{-1}
s	The surface area of the leaf inside the cuvette	m^2
P	Atmospheric pressure	bar
T_{leaf}	Leaf temperature	^{o}C
$g_{s(H2O)}$	Stomatal conductance for water vapour	$\frac{g^t_{H_2O}\times g_{b(H_2O)}}{g_{b(H_2O)}-g^t_{H_2O}}$
$g_{b(H2O)}$	Boundary layer conductance for water vapour	Calibrated for the cuvette we used
g^t_{H2O}	Conductance for water vapor through the boundary layer and stomata	$E\left(\frac{1-\left(\frac{w_i+w_a}{2}\right)}{w_i-w_a}\right)$, mol m^{-2}s^{-1}
g_s	Stomatal conductance for CO_2	$\frac{g_{s(H_2O)}}{1.6}$
g_b	Boundary conductance for CO_2	$\frac{g_{b(H_2O)}}{1.37}$
g^t_{CO2}	Conductance for CO_2 through the boundary layer and stomata	$\frac{g_s\times g_b}{g_s+g_b}$
Γ^*	CO_2 compensation point	45 µmol m^{-2}s^{-1}
g_{m13}	CO_2 conductance from intercellular air space to the site of carboxylation calculated using $\Delta_A{}^{13}C$ (for C_3 plants only)	mol m^{-2}s^{-1}bar^{-1}
g_{m18}	CO_2 conductance from intercellular air space to CO_2-H_2O exchange site calculated using $\Delta_A{}^{18}O$	mol m^{-2}s^{-1}bar^{-1}
c_i	Mole fraction of CO_2 in the intercellular air space	$\frac{\left(g^t_{CO_2}-\frac{E}{2}\right)c_a-A_n}{\left(g^t_{CO_2}+\frac{E}{2}\right)}$ mol mol^{-1}
c_s	Mole fraction of CO_2 at the leaf surface	$c_a - \frac{A_n}{g_b}$
c_m	Mole fraction of CO_2 at the site of CO_2-H_2O exchange	mol mol^{-1}
c_c	Mesophyll conductance to the chloroplast (for C_3 plants)	$c_i - \frac{A_n}{g_{m13}}$ mol mol^{-1}
t^{13}	Ternary correction for $^{13}CO_2$	$\frac{(1+a_{13bs})E}{2g^t_{CO_2}}$
t^{18}	Ternary correction for $C^{18}OO$	$\frac{(1+a_{18bs})E}{2g^t_{CO_2}}$
t^{17}	Ternary correction for $C^{17}OO$	$\frac{(1+a_{17bs})E}{2g^t_{CO_2}}$
R	Dark respiration rate	0.8 µmol m^{-2}s^{-1}
R_D	Day respiration rate	0.5 × R µmol m^{-2}s^{-1}
**Oxygen and carbon isotope effects**		
$\varepsilon^{18}{}_k$	Kinetic fractionation of water vapour in the air	$\frac{28g_b+19g_s}{g_b+g_s}$, ‰
$\varepsilon^{18}{}_{equ}$	Equilibrium fractionation between liquid and gas phase of water vapor	$2.644 - 3.206\left(\frac{10^3}{T_{leaf}}\right) + 1.534\left(\frac{10^6}{T_{leaf}}\right)$, ‰
a_{13bs}	Weighted fractionation for ^{13}COO as CO_2 diffuses through the boundary layer and stomata	$\frac{(c_s-c_i)a_{13s}+(c_a-c_s)a_{13b}}{c_a-c_i}$ ‰
a_{18bs}	Weighted fractionation for $C^{18}OO$ as CO_2 diffuses through the boundary layer and stomata	$\frac{(c_s-c_i)a_{18s}+(c_a-c_s)a_{18b}}{c_a-c_i}$ ‰
a_{13bs}	Weighted fractionation for ^{13}COO as CO_2 diffuses through the boundary layer and stomata	$\frac{(c_s-c_i)a_{13s}+(c_a-c_s)a_{13b}}{c_a-c_i}$, ‰
a_{18bs}	Weighted fractionation for $C^{18}OO$ as CO_2 diffuses through the boundary layer and stomata	$\frac{(c_s-c_i)a_{18s}+(c_a-c_s)a_{18b}}{c_a-c_i}$, ‰
$\overline{a}_{18}$	Weighted fractionation of $C^{18}OO$ as it diffuses through the boundary layer, stomata and liquid phase in series	$\frac{(c_i-c_m)a_{18w}+(c_s-c_i)a_{18s}+(c_a-c_s)a_{18b}}{c_a-c_m}$, ‰
a_{13b}	Fractionation in $^{13}CO_2$ as CO_2 diffuses through the boundary layer	2.9 ‰

a_{13s}	Fractionation in $^{13}CO_2$ as CO_2 diffuses through the stomata	4.4 ‰
a_m	Fractionation factor for dissolution and diffusion through water	1.8 ‰
f	Fractionation factor for photorespiration (decarboxylation of glycine)	16 ‰
e	Fractionation factor for mitochondrial respiration	In this study we assumed zero fractionation
e'	Fractionation factor for mitochondrial respiration including the apparent fractionation	$e - e^*$
e^*	Apparent fractionation for day respiration, when $\delta^{13}C$ of CO_2 assimilated is different from that of the respiratory substrate	$\delta^{13}C_{tank} - \delta^{13}C_{growth}$ where $\delta^{13}C_{growth} = -8$ ‰
b	Fractionation factor for uptake by RubisCO	29 ‰
α_f	Fractionation due to photorespiration (decarboxylation of glycine)	$1+f$
α_e	Fractionation due to day respiration	$1+e$
α_b	Fractionation due to uptake by RubisCO	$1+b$
a_{17b}	Fractionation of $C^{17}OO$ as CO_2 diffuses through the boundary layer	2.9 ‰
a_{17s}	Fractionation in $C^{17}OO$ as CO_2 diffuses through stomata	4.4 ‰
a_{18b}	Fractionation of $C^{18}OO$ as CO_2 diffuses through the boundary layer	5.8 ‰
a_{18s}	Fractionation in $C^{18}OO$ as CO_2 diffuses through stomata	8.8 ‰
a_{17w}	Fractionation in $C^{17}OO$ due to diffusion and dissolution in water	0.382 ‰
a_{18w}	Fractionation in $C^{18}OO$ due to diffusion and dissolution in water	0.8 ‰
$\varepsilon^{18}{}_W$	Equilibrium fractionation of CO_2 and water for $C^{18}OO$	$\frac{17604}{T_{leaf}} - 17.93$
$\varepsilon^{18}{}_k$	kinetic fractionation of water vapor in air	$\frac{28 \times g_b + 19 \times g_s}{g_b + g_s}$
$\varepsilon^{18}{}_{equ}$	equilibrium fractionation between the liquid and gas phase water	$2.644 - 3.206 \times (\frac{10^3}{T}) + 1.534 \times (\frac{10^6}{T})$
**Isotopic composition**		
$\delta^{18}O_A$	$\delta^{18}O$ of the assimilated CO_2	$\frac{\delta^{18}O_a - \Delta_A{}^{18}O}{\Delta_A{}^{18}O+1} = \delta^{18}O_a - \frac{c_e}{c_e - c_a}(\delta^{18}O_a - \delta^{18}O_e)$
$\delta^{18}O_{io}$	$\delta^{18}O$ of CO_2 in the intercellular air space ignoring ternary correction	$\delta^{18}O_A\left(1 - \frac{c_a}{c_i}\right)(1 + a_{18bs}) + \frac{c_a}{c_i}(\delta^{18}O_a - a_{18bs}) + a_{18bs}$
$\delta^{18}O_i$	$\delta^{18}O$ of CO_2 in the intercellular air space	$\frac{\delta^{18}O_{io} + t^{18}\left(\delta^{18}O_A\left(\frac{c_a}{c_i}+1\right) - \delta^{18}O_a\frac{c_a}{c_i}\right)}{1+t^{18}}$
$\delta^{18}O_m$	$\delta^{18}O$ of CO_2 at the site of CO_2-H_2O exchange	$(\delta^{18}O_{bl} + 1) \times (1 + \varepsilon^{18}_W) - 1$
$\delta^{13}C_{substrate}$	Isotope (^{13}C) ratio of substrate used for dark respiration	$\frac{\delta^{13}C_a - \Delta_A{}^{13}C}{\Delta_A{}^{13}C+1}$
$\Delta_A{}^{13}C_{obs}$	^{13}C-photosynthetic discrimination	$\frac{\zeta(\delta^{13}C_a - \delta^{13}C_e)}{1+\delta^{13}C_a - \zeta(\delta^{13}C_a - \delta^{13}C_e)}$, ‰
$\Delta_A{}^{18}O_{obs}$	^{18}O-photosynthetic discrimination	$\frac{\zeta(\delta^{18}O_a - \delta^{18}O_e)}{1+\delta^{18}O_a - \zeta(\delta^{18}O_a - \delta^{18}O_e)}$, ‰
$\Delta_A{}^{13}C_{obs}$	^{13}C-photosynthetic discrimination (Farquhar model)	$\left(\frac{1}{1-t}\right)\left[a_{13b}\frac{c_a - c_s}{c_a} + a_{13s}\frac{c_s - c_i}{c_a}\right] + \left(\frac{1+t}{1-t}\right)\left[a_m\frac{c_i - c_c}{c_a} + b\frac{c_c}{c_a} - \frac{\alpha_b}{\alpha_e\alpha_R}e\frac{R_D}{A}\frac{c_c}{c_a} - \frac{\alpha_b}{\alpha_f\alpha_R}f\frac{\Gamma^*}{c_a}\right]$
$\Delta_A{}^{13}C_i$	^{13}C-photosynthetic discrimination (assuming no mesophyll conductance, i.e $c_i = c_c$)	$\left(\frac{1}{1-t}\right)\left[a_{13b}\frac{c_a - c_s}{c_a} + a_{13s}\frac{c_s - c_i}{c_a}\right] + \left(\frac{1+t}{1-t}\right)\left[b\frac{c_c}{c_a} - \frac{\alpha_b}{\alpha_e\alpha_R}e\frac{R_D}{A}\frac{c_c}{c_a} - \frac{\alpha_b}{\alpha_f\alpha_R}f\frac{\Gamma^*}{c_a}\right]$
$\Delta_A{}^{18}O_{obs}$	^{18}O-photosynthetic discrimination	$\frac{\zeta(\delta^{18}O_a - d^{18}O_e)}{1+\delta^{18}O_a - \zeta(\delta^{18}O_a - \delta^{18}O_e)}$, ‰
$\Delta_A{}^{18}O_{FM}$	Farquhar model for ^{18}O-photosynthetic discrimination	$\frac{\bar{a}_{18} + \frac{c_m}{c_a - c_m}(1+t^{18})\,\delta^{18}O_{ma} + \bar{a}_{18}t^{18}\frac{c_a - c_m}{c_a - c_m}}{1 - \frac{c_m}{c_a - c_m}(1+t^{18})\delta^{18}O_{ma} - t^{18}\frac{c_a + c_m}{c_a - c_m}}$
$\delta^{18}O_e$	$\delta^{18}O$ of CO_2 entering the cuvette	Measured
$\delta^{18}O_a$	$\delta^{18}O$ of CO_2 leaving the cuvette	Measured
$\delta^{18}O_{ma}$	$\delta^{18}O$ of CO_2 equilibrated with the leaf water at the evaporating site relative to the CO_2 leaving the cuvette	$\frac{\delta^{18}O_m - \delta^{18}O_a}{1 - d^{18}O_a}$
$\Delta_A\delta^{47}{}_{obs}$	δ^{47}-photosynthetic discrimination	$\frac{\zeta(\delta^{47}{}_a - \delta^{47}{}_e)}{1 + \delta^*{}_a - \zeta(\delta^{47}{}_a - \delta^{47}{}_e)}$
Δ_{47}	The anomaly of mass 47 isotopologue relative to the abundance expected from a random distribution	$\left[\left(\frac{^{47}R}{^{47}R^*} - 1\right)\right]$

$\Delta_A\Delta_{47obs}$	Δ_{47}- photosynthetic discrimination	$\frac{\zeta\,(\Delta_{47a} - \Delta_{47e})}{1 + \Delta_{47a} - \zeta(\Delta_{47a} - \Delta_{47e})}$

Table S2: Isotopic composition of synthetic air spiked with scrambled CO_2 that was used for the leaf chamber experiments.

	$\delta^{13}C$ VPDB [‰]	$\delta^{18}O$ VSMOW [‰]	Δ_{47} [‰]
**Cylinder I** *(-IX)*	-2.47 ± 0.05	25.5 ± 0.3	0.19 ± 0.05
**Cylinder I (via empty cuvette)**	-2.49 ± 0.05	24.7 ± 0.3	0.24 ± 0.05
	-2.47 ± 0.05	25.0 ± 0.3	0.16 ± 0.05
	-2.50 ± 0.05	25.3 ± 0.3	0.20 ± 0.05
	-2.55 ± 0.05	25.5 ± 0.3	0.26 ± 0.05
	-2.41 ± 0.05	25.1 ± 0.3	0.31 ± 0.05
	-2.60 ± 0.05	25.3 ± 0.3	0.23 ± 0.05
	-2.53 ± 0.05	25.7 ± 0.3	0.31 ± 0.05
Mean ± SD	*-2.50* ± 0.06	*25.3* ± 0.3	*0.24* ± 0.05
**Cylinder II** *(-X)*	-2.51 ± 0.08	25.1 ± 0.3	0.07 ± 0.05
**Cylinder II (via empty cuvette)**	-2.39 ± 0.08	25.7 ± 0.3	0.17 ± 0.05
	-2.32 ± 0.08	25.4 ± 0.3	0.08 ± 0.05
	-2.45 ± 0.08	26.1 ± 0.3	0.02 ± 0.05
	-2.49 ± 0.08	25.1 ± 0.3	0.01 ±0 .05
Mean ± SD	*-2.43* ± 0.07	*25.5* ± 0.4	*0.07* ± 0.06

References:

1 Busch, F. A., Holloway-Phillips, M., Stuart-Williams, H. & Farquhar, G. D. Revisiting carbon isotope discrimination in C_3 plants shows respiration rules when photosynthesis is low. *Nat. Plants* **6**, 245-258 (2020).

2 Farquhar, G. D. & Cernusak, L. A. Ternary effects on the gas exchange of isotopologues of carbon dioxide. *Plant Cell Environ* **35**, 1221-1231, doi:10.1111/j.1365-3040.2012.02484.x (2012).

3 Gillon, J. S. & Yakir, D. Internal conductance to CO_2 diffusion and $C^{18}OO$ discrimination in C_3 leaves. *Plant Physiol.* **123**, 13 (2000).

4 Cernusak, L. A., Farquhar, G. D., Wong, S. C. & Stuart-Williams, H. Measurement and interpretation of the oxygen isotope composition of carbon dioxide respired by leaves in the dark *Plant Physiology* **136**, 13 (2004).

5 Barbour, M. M., Evans, J. R., Simonin, K. A. & von Caemmerer, S. Online CO_2 and H_2O oxygen isotope fractionation allows estimation of mesophyll conductance in C_4 plants, and reveals that mesophyll conductance decreases as leaves age in both C_4 and C_3 plants. *New Phytol.*, 875-889 (2016).

6 Holloway-Phillips, M., Cernusak, L. A., Stuart-Williams, H., Ubierna, N. & Farquhar, G. D. Two-source $\delta^{18}O$ method to validate the $CO^{18}O$-photosynthetic discrimination model: Implications for mesophyll conductance. *Plant Physiology* **181**, 15 (2019).

7 Osborn, H. L. *et al.* Effects of reduced carbonic anhydrase activity on CO_2 assimilation rates in Setaria viridis: a transgenic analysis. *J. Exp. Bot.* **68**, 11 (2017).
